# Lake Avernus Has Turned Red: Bioindicator Monitoring Unveils the Secrets of “Gates of Hades”

**DOI:** 10.3390/toxins15120698

**Published:** 2023-12-13

**Authors:** Germana Esposito, Evgenia Glukhov, William H. Gerwick, Gabriele Medio, Roberta Teta, Massimiliano Lega, Valeria Costantino

**Affiliations:** 1 The Blue Chemistry Lab, Department of Pharmacy, University of Naples Federico II, 80131 Napoli, Italy; germana.esposito@unina.it (G.E.); valeria.costantino@unina.it (V.C.); 2 Center for Marine Biotechnology and Biomedicine, Scripps Institution of Oceanography, and Skaggs School of Pharmacy and Pharmaceutical Sciences, University of California San Diego, La Jolla, CA 92093, USA; eglukhov@ucsd.edu (E.G.); wgerwick@health.ucsd.edu (W.H.G.); 3 Department of Engineering, University of Naples Parthenope, 80133 Napoli, Italy; gabriele.medio@uniparthenope.it

**Keywords:** cyanobacterial bloom, cyanotoxins, molecular networking, remote sensing, bioindicators, anabenopeptins

## Abstract

Lake Avernus is a volcanic lake located in southern Italy. Since ancient times, it has inspired numerous myths and legends due to the occurrence of singular phenomena, such as coloring events. Only recently has an explanation been found for them, i.e., the recurring color change over time is due to the alternation of cyanobacterial blooms that are a consequence of natural nutrient inputs as well as pollution resulting from human activities. This current report specifically describes the red coloring event that occurred on Lake Avernus in March 2022, the springtime season in this region of Italy. Our innovative multidisciplinary approach, the ‘Fast Detection Strategy’ (FDS), was devised to monitor cyanobacterial blooms and their toxins. It integrates remote sensing data from satellites and drones, on-site sampling, and analytical/bioinformatics analyses into a cohesive information flow. Thanks to FDS, we determined that the red color was attributable to a bloom of *Planktothrix rubescens*, a toxin-producing cyanobacterium. Here, we report the detection and identification of 14 anabenopeptins from this *P. rubescens* strain, seven of which are known and seven are newly reported herein. Moreover, we explored the mechanisms and causes behind this cyclic phenomenon, confirming cyanobacteria’s role as reliable indicators of environmental changes. This investigation further validates FDS’s effectiveness in detecting and characterizing cyanobacterial blooms and their associated toxins, expanding its potential applications.

## 1. Introduction

Advances in science and research over the last century have provided explanations for many myths and legends of the past, as well as a better understanding of the consequences and impacts of human activities on the environment. Recently, this improved awareness has spurred the scientific community to develop multidisciplinary techniques for the analysis of environmental “anomalies”; for example, those based on the monitoring of bioindicators as sentinels of pollution [1,2].

In the context of our ongoing monitoring project of internal and coastal waters, aimed at early detection and early warning of cyanobacterial blooms and associated cyanotoxins, using our advanced multidisciplinary protocol termed ‘Fast Detection Strategy’ (FDS) [3], we caught the onset of a bloom that occurred on Lake Avernus in March 2022, the springtime season in Italy, when the lake suddenly turned red.

Since ancient times, Lake Avernus in southern Italy has inspired numerous myths and legends due to this unique phenomenon, such as color variations. Lake Avernus (Lago d’Averno) is quite small and located in a volcanic area known as the Phlegrean Fields (Campi Flegrei), close to Naples (Italy). An old legend states that Lake Avernus was the “*Gate of Hades*”. Here, Dante located the entrance to the underworld, and, in even more remote times, its waters were said to have been the scene of the clash between Zeus and the Titans. Lake Avernus has inspired poets and writers for centuries with its evocative and restless landscape. 

The stories concerning this lake were not only the product of creativity and imagination but also the result of attempts to explain unique phenomena that were not understood in ancient and even modern times. For example, several times in the past, it has been observed that entire flocks of birds have died while flying over the lake and fell into it as a group. A similar fate was reported for an entire school of fish. The resulting myth has been that the “Gates of Hades” bring death to anyone who gets too close. Centuries later, it has become clear that natural toxic gasses from this volcanic lake have caused these mass die-off events of fish and birds [4].

**Figure 1:**
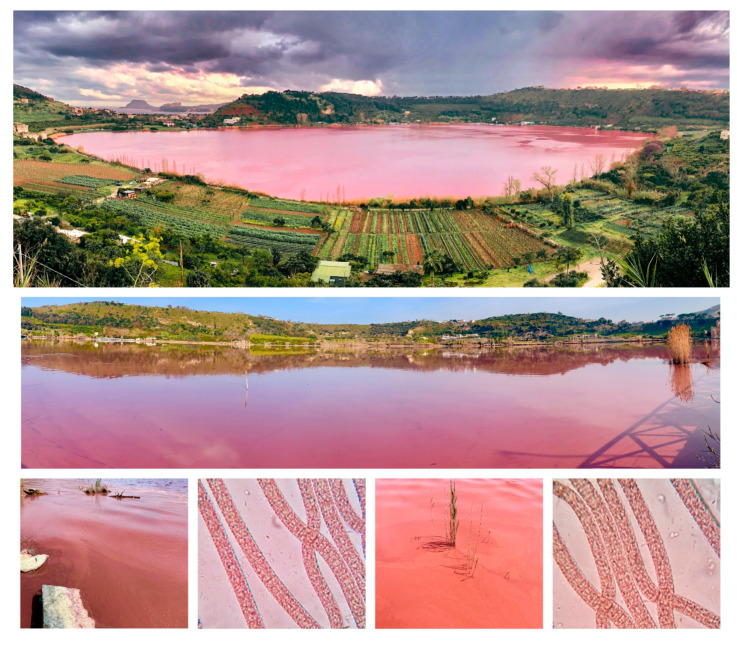
Picture of Lake Avernus 2022 red bloom and microscopic observation of its waters.

During the last decades, the lake has experienced another extraordinary phenomenon, namely periodic and significant changes in the color of the water. Our previous research has shown that these color changes are linked to the alternating blooms of various species of cyanobacteria [5,6]. The current work introduces the results of our research on a coloring event that occurred on Lake Avernus in March 2022, the springtime season in Italy, when the lake turned a brilliant red color (Figure 1): apparently the “*Gate of Hades*” had opened again. 

Tackling this intricate phenomenon, and indeed any environmental challenge, requires adopting a multidisciplinary approach. Given that the environment functions as a complex system, a single discipline itself cannot provide a comprehensive understanding of the phenomenon. 

Using our FDS strategy [3], which combines remote and proximal sensing data analysis (e.g., by satellite, drone, and on-site sampling) alongside analytical/bioinformatics analyses, the red color event was successfully characterized as a bloom of *Planktothrix rubescens* [7], a red-pigmented cyanobacterium that produces a series of cyanotoxins, such as anabaenopeptins [8].

The anabaenopeptins are a class of cyclic hexapeptides, first reported from a culture of *Anabaena flos-aquae* NRC 525-17, in which an exocyclic amino acid is connected to the pentapeptide ring structure through an ureido linkage. The ureido linkage and the Lys residue responsible for the ring formation are conserved motifs within the anabaenopeptins, while the structural variability is created by different amino acids in the remaining positions [9]. As a part of the FDS, HRMS/MS-based molecular networking was used to profile the metabolome of the bloom sample, allowing the detection and identification of several known and new anabaenopeptin variants.

Given the correlation between the cyanobacterial bloom and the environmental status of the lake (i.e., eutrophication) [6], the possible causes that led to this recurring phenomenon on Lake Avernus have also been investigated [10].

## 2. Results

### 2.1. Remote/Proximal Sensing and Analysis of Multispectral Data

**Figure 2:**
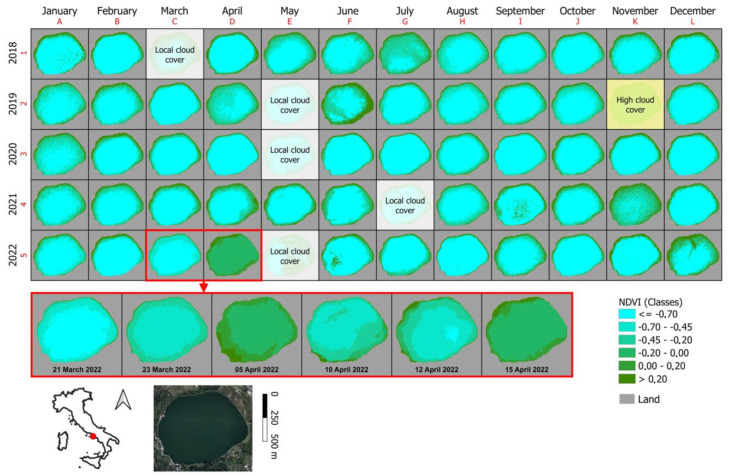
Monitoring of the multispectral response of the waters of Lake Avernus using the NDVI index.

Processing of the satellite acquisitions with the NDVI index [2,11] over the time range of 2018 to 2022 (Figure 2) revealed an anomaly in the multispectral response in the months of March–April 2022, a period corresponding to a distinctively red color of the lake.

After conducting this analysis, and given the particular color of the water, it was deemed more appropriate to use the NDTI index [12] for increased accuracy of the multispectral analyses because it is more consistent with the reddish chromatic variations of the water. Due to the imperfect correlation of the data obtained from the two different indices and considering the inability to use satellite data in the visible range because the lake was visualized as a dark surface (as shown in the miniature inset at the bottom of Figure 2), it was necessary to acquire proximal sensing data.

**Figure 3:**
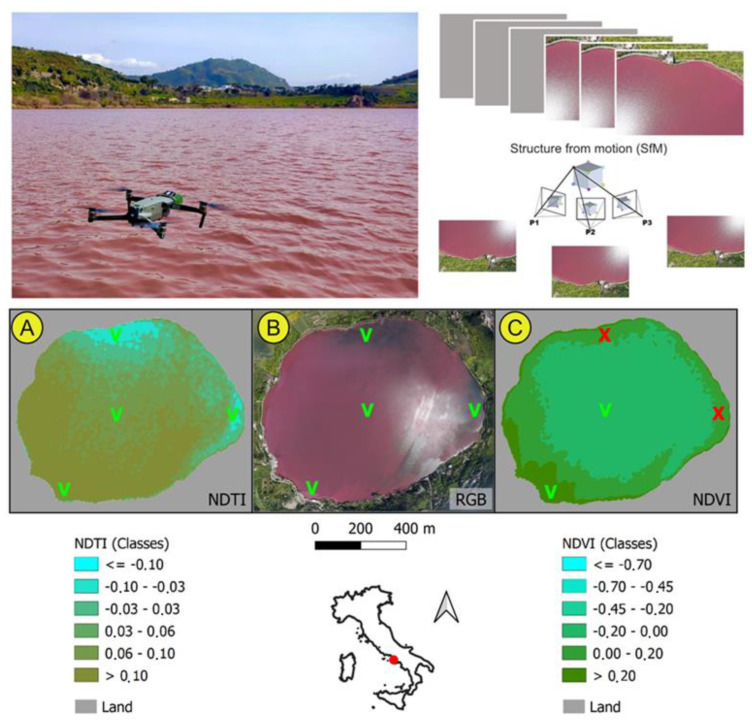
Calibration of remote (satellite) acquisitions on the basis of proximal ones using UAV/drone and processing data with Structure from Motion (SfM) software to create a reference global view. Orthomosaic of RGB data of Lake Avernus acquired by drone (**B**) and NDVI (**C**) and NDTI (**A**) indices based on satellite data. V and X are check-points: V stands for verified and confirmed by both indices; X are the spots where NDVI data are not coherent with RGB and NDTI values.

Hundreds of pictures were acquired by an UAV/drone, and these were processed with Structure from Motion (SfM) software to create a reference global view (Figure 3). The proximal sensing drone data, dating back to 5 April 2022, were used to validate and calibrate the satellite remote sensing data from NDVI (Figure 3a) and NDTI (Figure 3c). 

The orthomosaic of the entire surface of Lake Avernus (Figure 3b) by drone-enabled observation shows that the reddish color of the water tends to be quite homogeneous in the center of the lake, relatively negligible in the north and east, and very intense in the south-west.

**Figure 4:**
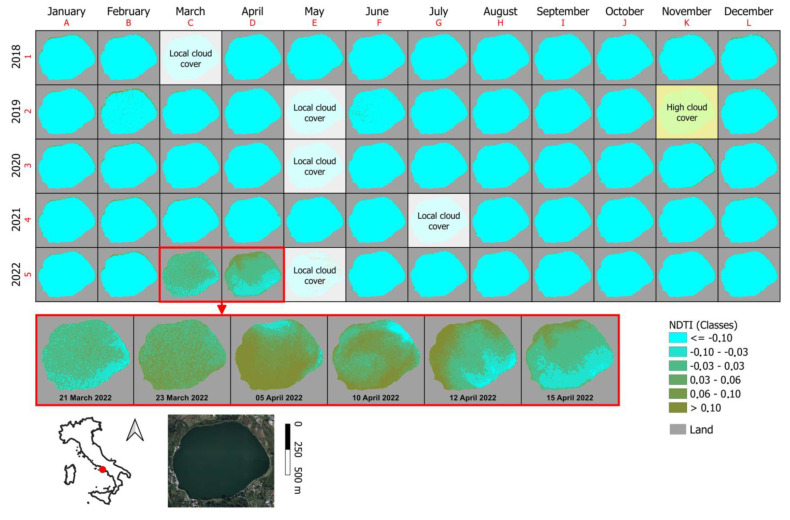
Monitoring of the multispectral response of the waters of Lake Avernus using the NDTI index over a timespan between January 2018 and December 2022.

By reprocessing the satellite acquisitions using the NDTI index (Figure 4) and defining a classification of the index values, only the multispectral anomalies of interest, i.e., the 2022 reddish coloration of the lake waters, were emphasized.

**Figure 5:**
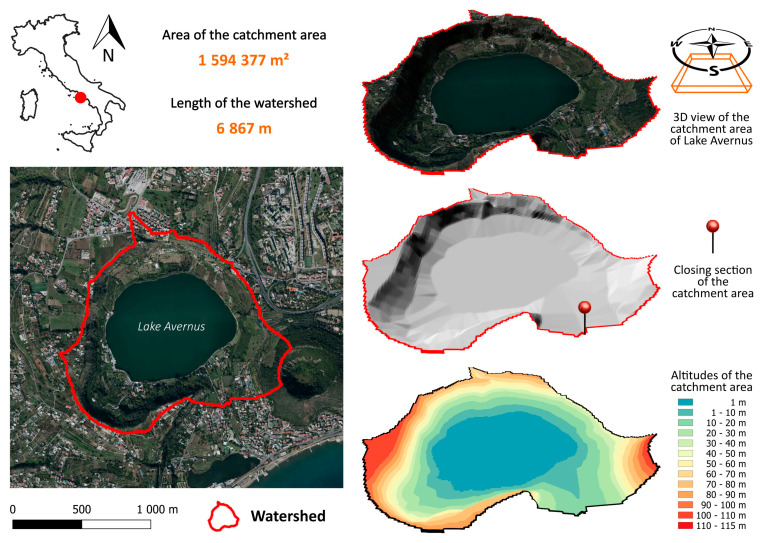
Catchment area of Lake Avernus.

To investigate the cause-and-effect mechanisms responsible for the anomalous reddish coloration of the waters of Lake Avernus and whether this phenomenon was connected to any pollution of the surface waters, we examined the catchment area of Lake Avernus for a given closure section. This analysis of the watershed allowed a determination of the area within which surface water is collected and conveyed into the lake. This analysis was conducted on the digital elevation model (DEM) called TINITALY/01 (also known as DEM “TINITALY”) [13]. In particular, the catchment area of interest (Figure 5) was determined through the QGIS software (3.8.1, https://www.qgis.org/, accessed on 15 April 2022) using the functions present in the processing tools.

**Figure 6:**
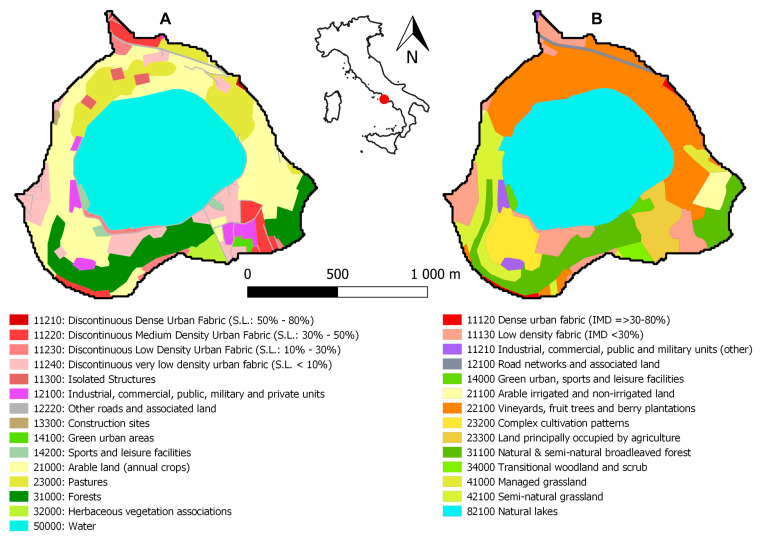
Thematic maps of land cover and land use in the Lake Avernus catchment area. Thematic maps were obtained from the Copernicus Land Monitoring Service (CLMS), jointly implemented by the European Environment Agency (EEA) and the European Commission DG Joint Research Centre (JRC): (**A**) Urban Atlas 2018; (**B**) Coastal Zones 2018.

In addition, we also considered possible anthropic activities in this area that could be responsible for the release of pollutants and therefore impact the lake through surface runoff of rainwater as well as other water inputs. To understand potential sources of pollution within the basin [14], we employed information on land cover and utilization provided by the Copernicus Program. This used thematic maps (Figure 6) from the Copernicus Land Monitoring Service (CLMS), jointly implemented by the European Environment Agency (EEA) and the European Commission DG Joint Research Centre (JRC).

This analysis revealed that the main anthropic activities on the lake that could originate the release of pollutants/nutrients through the catchment area are intensive agriculture (vineyards, fruit trees, etc.), natural and seminatural broadleaved forests, commercial units, and medium-to-low-density urban areas.

### 2.2. Taxonomic Analysis of Prevalent Cyanobacteria

Alerted and driven by remote/proximal sensing, we collected an unfiltered 1 L sample of 2022 Lake Avernus water along its north shore on 30 March 2022. The identity of the microbial population comprising the bloom was determined through a combination of microscopic observation and 16S rRNA metagenomic analysis [2]. The light microscope analysis (Figure 1) revealed the presence of a red-colored cyanobacterium with morphological features consistent with *Planktothrix rubescens* [15] (straight trichomes without sheath, red/purple in color, cylindrical cells with a diameter of 6–8 µm, numerous aerotopes, and densely granulated). 

The metagenomic DNA extracted from the collected sample was used as a template for PCR amplification with 16S rRNA universal and cyanobacterial-specific primers. The purified PCR products were sequenced and found to be identical at a 99% sequence identity threshold with *Planktothrix rubescens*. This was further confirmed by a best match of sequences from a BLASTn search and the neighboring joining tree of the 16S cyanobacterial rRNA fragments (Supplementary Material). Therefore, the *P. rubescences* strain (here named AvL22) is the predominant cyanobacterial species present in the 2022 Lake Avernus bloom. No other strains were noticed either under a microscope or revealed by 16S rRNA sequencing.

### 2.3. Molecular Networking and Identification of Anabaenopeptin Variants

The methanol extract of the 2022 red bloom sample was profiled using MS^2^-based molecular networking obtained from liquid chromatography high-resolution tandem mass spectrometry (LC-HRMS/MS) data (https://gnps.ucsd.edu/ProteoSAFe/status.jsp?task=dab305d4d24e4123bb455a0144da2ba1, accessed on 4 November 2022) [16]. The resulting network was composed of 291 nodes, of which 60 were grouped into 17 clusters and 231 self-loops. In order to dereplicate known peptidic and non-peptidic natural products, the Dereplicator+ tool on GNPS was used [17,18]. 

**Figure 7:**
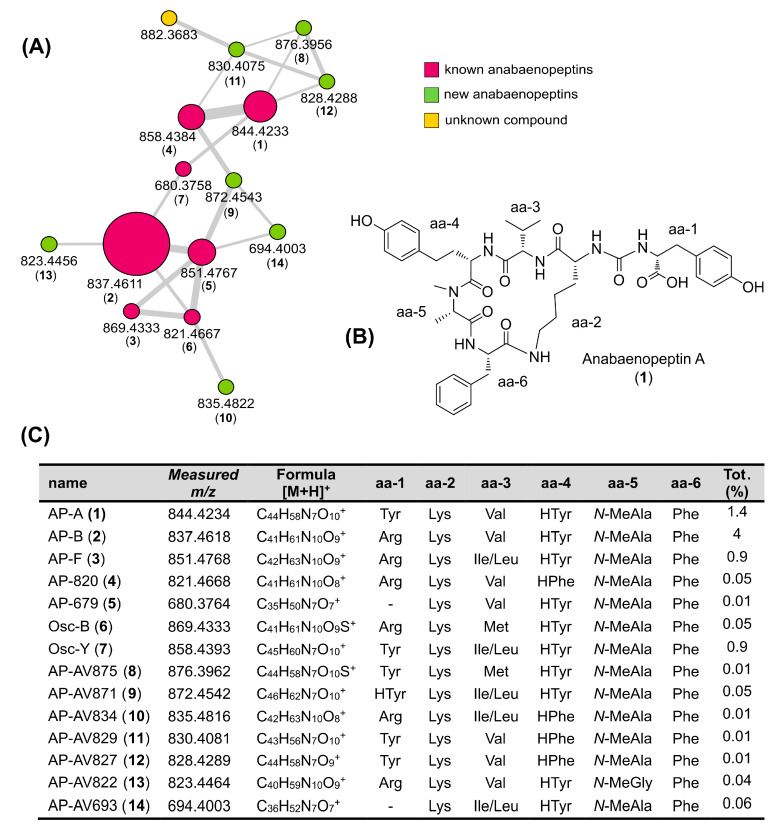
(**A**) Anabaenopeptin cluster: nodes are labeled with the parent mass; node size is proportional to metabolite amounts (precursor ion intensity); and the color of each node reflects annotation (known anabaenopeptins are colored in purple, new anabaenopeptins in green, and unknown compounds in yellow). Edge thickness is related to cosine score similarity. (**B**) Structure of anabaenopeptin A. (**C**) Anabaenopeptin variants from the Lake Avernus 2022 bloom: % refers to the percentage abundance calculated for the entire extract.

This allowed annotation of four nodes belonging to the largest (15 nodes) cluster of the network (Figure 7), as anabaenopeptins A (**1**, *m*/*z* 844.4234), B (**2**, *m*/*z* 837.4611), oscillamides B (**3**, 869.4333), and Y (**4**, *m*/*z* 858.4393). The cluster was therefore assigned to the chemical class of anabaenopeptins, cyclic hexapeptides composed of five amino residues connected through an ureido linkage to an exocyclic amino acid [9]. The most abundant anabaenopeptin (B, compound **2**) was purified from the extract, and its structure was confirmed by comparison of its MS/MS spectra (Figure S4) and NMR data (Figures S17–S19 and Table S1) with those reported [19]. The HRMS spectrum of compound **2** showed, in addition to the [M + H]^+^ ion at *m*/*z* 837.4611 (the base peak), an intense doubly-charged ion [M + 2H]^2+^ at *m*/*z* 419.2344, corresponding to the molecular formula C_41_H_60_N_10_O_9_.

**Figure 8:**
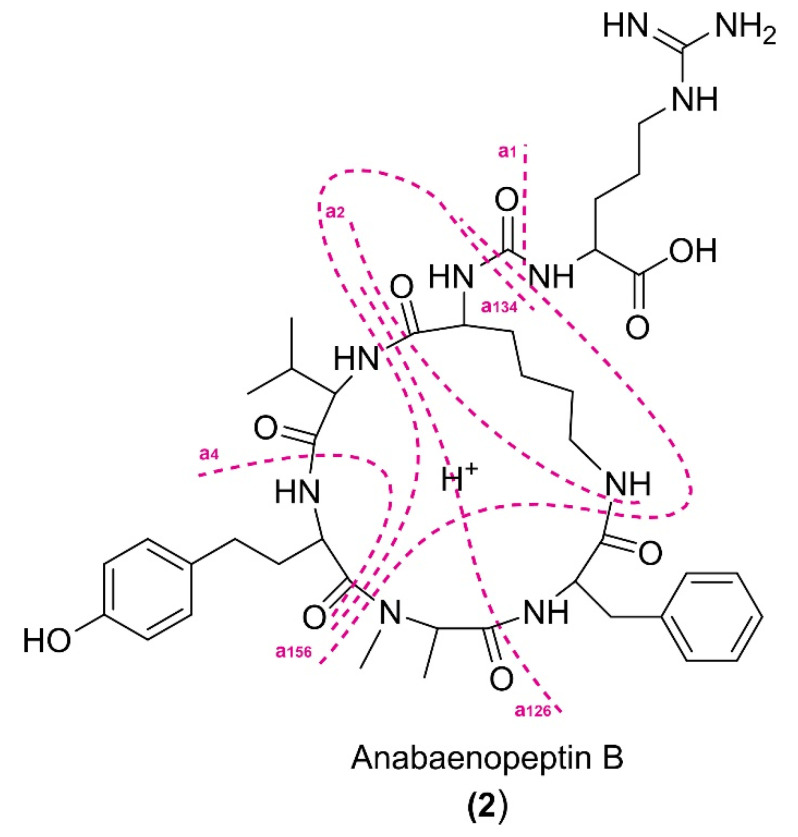
Fragmentation pattern of anabaenopeptin B (**2**).

The high-resolution MS/MS fragmentation pattern of 2 (Figure 8) was then used as a model by which to compare with all the anabaenopeptin variants of the cluster and to identify seven new congeners. The assignment of the principal fragment ions of the known and new anabaenopeptins is shown in Table 1. 

As typical for cyclic peptides [20], most fragment ions derive from the cleavage of two amide bonds, with the loss of one (e.g., a_1_ from the loss of aa-1), two (e.g., a_14_ due to the loss of aa-1 and aa-4), or three (a_134_) amino acid residues from the parent ion [21]. 

In the HRMS/MS spectrum of **2**, the fragment ion (base peak) at *m*/*z* 637.3700 (a_1_-CO, C_34_H_49_N_6_O_6_^+^), due to the neutral loss of 200.0918 amu (C_7_H_12_O_3_N_4_) from the parent ion (proton transfer to the charged species), confirmed the presence of a ureido-linked arginine residue in the side chain (aa-1). The same fragment a_1_-CO was also present in the MS/MS spectra of **1**, suggesting that the two compounds share the same pentapeptidic ring core, and therefore their difference lies in the exocyclic amino acid residue. Correspondingly, from anabaenopeptin A (**1**), there is a loss of tyrosine (loss of 207.0535 amu, C_9_H_9_O_3_N + CO from the parent ion). Sequential amino acid cleavages from a_1_-CO yielded a series of diagnostic fragment ions shared between the two compounds at *m*/*z* 509.2753 (a_2_), 460.2914 (a_14_-CO), 362.2073 (a_126_), 379.2346 (a_134_ + H_2_O), and 387.2386 (a_156_), due to the loss of Lys, HTyr, and the dipeptidic residues Lys-Phe, Val-HTyr, and *N*-MeAla-Phe, respectively. These fragment ion masses confirmed the structure of the pentapeptidic ring as cyclo (Lys-Val-HTyr-*N*-MeAla-Phe). 

Oscillamide B (**3**) differs from anabaenopeptin B (**2**) in the substitution of the Val residue (aa-3) with a Met residue. In the HRMS/MS spectrum of **3**, all of the ions originating from the loss of fragments containing aa-3 were in common with the corresponding ones in anabaenopeptin B (**2**), thus confirming the location of the substitution and the annotation afforded by Dereplicator+.

Oscillamide Y (**4**) was the final anabaenopeptin dereplicated by GNPS. Compound **4** contains an additional CH_2_ compared to anabaenopeptin A (**1**) due to the presence of Ile/Leu in place of Val (aa-3). The structure of **4** was confirmed by the presence of the same fragment ion a_3_ as observed for **1** (*m*/*z* 745.3556, C_39_H_49_N_6_O_9_^+^); this fragment derives from the loss of an Ile/Leu residue (113.0837 amu). 

A manual in-depth analysis of HRMS/MS spectra allowed identification of compounds **5** and **6** as anabaenopeptin F and anabaenopeptin 820 [22], respectively. These latter two compounds were not annotated by Dereplicator+. Compound **5** (*m*/*z* 851.4767, C_42_H_63_N_10_O_9_^+^) showed an additional CH_2_ in comparison with anabaenopeptin B (**2**). In the HRMS/MS spectrum of **5**, the loss of an Ile/Leu-HTyr fragment (290.1627 amu, C_16_H_22_N_2_O_3_) was observed from the parent ion instead of a Val-HTyr fragment (276.1469 amu, C_15_H_20_N_2_O_3_), as occurs for compound **2**. This loss from compound **5** generates the same diagnostic ion a_34_, confirming the Val→Ile/Leu substitution at aa-3. Compound **6** (*m*/*z* 821.4668, C_41_H_61_N_10_O_8_^+^) lacks one oxygen atom in comparison with compound **2**. The fragment ion at *m*/*z* 460.2914 (a_14_-CO) in the HRMS/MS spectra of both compounds indicated that their difference lies in the amino acid residue aa-4; in compound **6**, this is a HPhe residue instead of the HTyr in compound **2**. 

For compound **7**, the fragment a_1_-CO at *m*/*z* 637.3727 (C_34_H_49_N_6_O_6_^+^) was observed. This is the same fragment mass as found in the HRMS/MS spectrum of **1** and **2**. However, for **7**, this results from a loss of 43.0058 amu (-CONH_2_) from the parent ion, thus indicating the lack of an amino acid linked to the ureido group and allowing identification of compound **7** as anabaenopeptin 679 [23].

Similarly, as for oscillamide B (**3**), the molecular formula of compound **8** (C_44_H_58_N_7_O_10_S^+^, *m*/*z* 876.3956) contained an additional sulfur atom compared to anabaenopeptin A (**1**), suggesting a Val→Met substitution at aa-3. This was confirmed by the HR-MS/MS spectrum of **8**, which contained a fragment ion at *m*/*z* 699.3160 (a_4_, C_34_H_47_N_6_O_8_S^+^), originating from the loss of a HTyr residue. However, this still differed by 32 amu from the corresponding a_4_ (*m*/*z* 667.3443, C_34_H_47_N_6_O_8_^+^) of anabaenopeptin A (**1**), and therefore, this fragment contained the extra sulfur atom. The same a_34_ (*m*/*z* 568.2762) ion was observed for both compounds. Substitution of methionine (or its oxidized form, methionine sulfoxide) for valine has previously been observed in several anabaenopeptins [24]; however, to the best of our knowledge, compound **8** (AP-AV875) has never been reported and is thus a new anabaenopeptin variant. 

Compound **9** (*m*/*z* 872.4543, C_46_H_62_N_7_O_10_^+^) possessed an additional CH_2_ compared with oscillamide Y (**4**). The same fragment ion at *m*/*z* 651.3853 (a_1_-CO, C_35_H_51_N_6_O_6_^+^) as for compound **4** is generated by the loss of 195.0907 amu (C_10_H_13_O_3_N) from the parent ion of **9**, and therefore indicates that **4** and **9** share the same ring core but differ in aa-1. Four different possibilities could explain the molecular formula of the aa-1 residue: *N*-MeTyr, HTyr, O-MeTyr, or Tyr-OMe; this latter possibility was confirmed on the basis of the loss of methanol (32.0275 amu) and methanol + CO (60.0203 amu) from the parent ion. In anabaenopeptin 871 [24], a residue of HTyr is in place of Phe (aa-6); therefore, compound **9,** namely AP-AV872, is a new anabaenopeptin variant.

The new congener **10** (*m*/*z* 835.4822, C_42_H_63_N_10_O_8_^+^, AP-AV834) lacked one O atom in comparison with **5**, suggesting a substitution at aa-4 of HTyr→HPhe. This was confirmed by the shared fragment ion a_4_ between compounds **5** and **10** (*m*/*z* 674.3908, C_32_H_52_N_9_O_7_^+^), due to the loss of a HPhe residue (161.0843 amu) from the parent ion for compound **10**. 

In comparison with **1**, compound **11** (*m*/*z* 830.4075, C_43_H_56_N_7_O_10_^+^; AP-AV829) lacked one CH_2_ due to a *N*-MeAla→Ala or *N*-MeGly substitution for aa-5. This was demonstrated by the presence of fragment ion a_5_ at *m*/*z* 759.3699 (C_40_H_51_N_6_O_9_^+^), which is in common with anabaenopeptin A (**1**) and is generated by the loss of Ala or N-MeGly residue (71.0373 amu, C_3_H_5_ON) from the parent ion of **11**. It was not possible to distinguish between the two isobaric amino acids; however, searches of the literature for either possibility resulted in no hits, and therefore compound **11** is a novel anabaenopeptin congener. 

Compound **12** (*m*/*z* 828.4288, C_44_H_58_N_7_O_9_^+^) lacked one O atom in comparison with **1**. The substitution HTyr→HPhe at aa-4 was confirmed from the relevant peak a_4_ due to the cleavage of the HPhe residue. Anabaenopeptin D and UIC827 [25] showed the same molecular formula as **12** but a different arrangement of amino acid residues; therefore, AP-AV827 (**12**) is a new compound.

When the Arg residue is lost from the parent ion of **13** (*m*/*z* 823.4456, C_40_H_59_N_10_O_9_^+^), the generated a_1_ ion and its correlated fragments (a_2_, a_126_) overlap with those deriving from **11**, suggesting the same new pentapeptidic ring as **11**, that is, cyclo (Lys-Val-HTyr-Ala/N-MeGly-Phe), but substituted with an Arg residue as the exocyclic amino acid. 

Compound **14** contains one CH_2_ group more than **7** and is therefore another example of a truncated anabaenopeptin lacking the side chain attached to the ureido group. The additional CH_2_ was located on aa-3 on the basis of the fragment ions a_1_, a_2_, and a_126_ that are in common with Osc-Y (**4**) and AP-AV871 (**9**), showing that an Ile/Leu residue was present at that position. AP-AV693 (**14**) is also a new compound.

## 3. Discussion and Conclusions

In the context of our monitoring program of Campania’s (Italy) coastal and inland waters, a sudden red bloom occurred in Lake Avernus in March 2022 and was detected and characterized through our FDS approach [3]. Our innovative strategy, drawing from multiple disciplines, harnesses various cutting-edge techniques and technologies for the full characterization of the phenomenon, otherwise unfeasible using each method individually. The effectiveness and robustness of this multidisciplinary approach have been previously tested and validated in several case studies [2,3] and further substantiated by the application of FDS to the 2022 Lake Avernus red bloom event. 

Indeed, it allowed not only early detection of the bloom but also the identification of the major species of the bloom (*Planktohrix rubescens*) as well as the identification of 14 variants of the anabaenopeptin toxins, seven of which are new anabaenopeptins. 

The use of MS-based molecular networking allowed a comprehensive profiling of the *P. rubescens* (AvL22) metabolome. These MS-based molecular identifications confirm the usefulness of this strategy for the fast dereplication of complex natural mixtures without the requirement for complex and time-consuming chromatographic purifications. 

The abundance of the main anabaenopeptin found in the Lake Avernus cyanobacterial extract, anabaenopeptin B (2), has been estimated to account for as much as 4% of the total extract. Given the well-known toxicity of the anabaenopeptins [26], concerns are raised about the serious risk to human and other animal safety. In addition to being a popular recreational location, the lake has an outlet to the sea, which is close to mussel farms, and therefore there is a risk that toxins can enter the food chain. A constant monitoring effort through FDS is currently the only way to provide a timely warning that a bloom event is occurring in Lake Avernus and thereby prevent potentially negative health and environmental consequences.

With this aim, the integration of remote and proximal sensing data within the FDS approach allowed for the onset of the cyanobacterial bloom as well as its periodic recurrence. 

Specifically, it was possible to observe that the anomalous reddish coloration of the lake waters occurring in the months of March–April 2022 was a rather singular event, even if not unique. This is especially notable given the time span that extends from 2018 to 2022. The last registered event dates back to 2007, as Ferranti et al. reported [5]. Therefore, Lake Avernus has been affected over time by various cyanobacterial blooms alternating between different species and associated toxins, demonstrating a possible relationship between cyanobacterial species and environmental conditions.

The dynamics and causes of these periodic blooms were also investigated. There are two broad areas of causation that could potentially lead to the recurring phenomenon of cyanobacteria blooming on Lake Avernus: (1) the favorable natural conditions associated with the volcanic origin of the lake, particularly those related to its physicochemical state and biogeochemical vertical structure [27]; and (2) the specific anthropic activities that release pollutants/nutrients into the lake through surface runoff of rainwater as well as non-rainwater.

The identification of the watershed catchment area of Lake Avernus allowed us to delimit the area from which surface water is collected and conveyed into the lake. Thematic maps of land cover and land use, provided by the Copernicus Program and defined by the closing section of the catchment area, revealed the main anthropic activities that release pollutants/nutrients as: intensive agriculture (vineyards, fruit trees, etc.), natural and seminatural broadleaved forests, commercial units, and medium-to-low density urban areas.

Although the full comprehension of the causes of cyanobacterial blooms in Lake Avernus, their alternation, and the production of toxins is still under study, constant monitoring, observation, and analysis enable us, time after time, to add a piece to the intricate and intriguing story of the “*Gates of Hades*”.

## 4. Materials and Methods

In recent years, Lake Avernus and its recurring cyanobacterial blooms have been the “test bed” of the FDS protocol [3]. This has been developed and optimized by our research groups with the hypothesis that cyanobacteria can be used as bioindicators of environmental pollution phenomena. This strategy is based on a multidisciplinary approach that involves multiple phases and multiple levels of data acquisition and analysis [28]. For the monitoring program of Lake Avernus, the following phases/data layers were used: (1) satellite remote sensing and analysis of related multispectral data; (2) drone proximal sensing and analysis of related multispectral data; (3) in-situ sampling and in-lab taxonomical identification; and (4) metabolomic profiling through HRMS/MS-based molecular networking [29].

### 4.1. Satellite Remote Sensing and Analysis of Related Multispectral Data

To understand the temporal evolution of the anomalies in Lake Avernus’ waters in terms of multispectral response, a monitoring by satellite survey was conducted over a period ranging from 2018 to 2022. Satellite acquisitions were processed with multispectral indices.

The satellite surveys employed the Sentinel-2 mission developed by the European Space Agency (ESA) within the Copernicus program. The Copernicus Sentinel-2 mission comprises a constellation of two polar-orbiting satellites placed in the same sun-synchronous orbit, phased at 180° to each other (https://sentinel.esa.int/web/sentinel/missions/sentinel-2, accessed on 30 March 2022) [30].

Among the outputs produced by the Copernicus mission, there is a collection of 100 × 100 km^2^ orthophotos in UTM/WGS84 projection related to different spectral bands. In particular, the orthophotos used in this study belong to the visible and near-infrared spectral bands and have a spatial resolution of 10 m. Thanks to the high temporal resolution (2–3 days at medium latitudes) of the satellite acquisitions, it was possible to obtain, for almost every month, at least one survey of Lake Avernus without cloud cover (Figure 2). The analysis of the multispectral response of the lake waters was initially conducted using the Normalized Difference Vegetation Index (NDVI). This multispectral index, first used by Rouse et al. [11], usually allows identification of the vegetated areas and their condition based on the following formula:

(1) NDVI = (R_NIR_ − R_RED_)/(R_NIR_ + R_RED_)

where R_RED_ and R_NIR_ are the reflectances of the red and near-infrared spectral bands, respectively.

Because the NDVI is very sensitive to chlorophyll, under certain conditions it also allows detection of the presence of algae and cyanobacterial blooms in the water; therefore, exploiting this peculiarity, we tried to understand, in the preliminary phase, if there were any potential anomalies of the multispectral response linked to these aquatic organisms. However, the NDVI proved not to be the most suitable index for describing, in multispectral terms, the particular phenomenon occurring on Lake Avernus. The original design of the NDVI did not envisage applications in water or multispectral responses characterized by high red reflectance.

As a result, to obtain more reliable information, we found it necessary to use the NDTI (Normalized Difference Turbidity Index), which combines the reflectance of the red (R_RED_) and green (R_GREEN_) spectral bands:

(2) NDTI = (R_RED_ − R_GREEN_)/(R_RED_ + R_GREEN_)

The NDTI provides important information on the turbidity of water bodies. As the turbidity increases, the value of this index increases. Since turbidity is linked to the dispersion and absorption of light caused by the presence of suspended particles, the presence of cyanobacterial blooms can strongly affect the NDTI index.

### 4.2. Drone Proximal Sensing and Analysis of Related Multispectral Data

The information deriving from satellite acquisitions allowed analysis of this phenomenon over a large area and time interval. However, remote sensing generally suffers from low spatial resolution, making satellite information unsuitable for detailed analysis. Furthermore, in many cases, remote sensing needs to be calibrated and validated using proximal information. Moreover, in conditions of high cloud cover, remote sensing is unsuitable for analyzing phenomena inside lake waters. For these reasons, a proximal sensing analysis based on data acquisition from a drone was also necessary. The drone employed for this study was a DJI Mavic 2 Pro equipped with a fully stabilized RGB camera capable of taking 20 megapixel aerial photos and a multispectral camera (MAPIR Survey 3, San Diego, CA USA) with an OCN (Orange, Ciano, NIR) filter.

To use the drone efficiently and safely, a flight mission was planned with the following points: the flight of the drone took place before the disappearance of the phenomenon of interest; the day of the mission coincided with the date of passage of one of the Sentinel 2 satellites over the place of interest; the day of the mission was characterized by good weather; the programmed flight path guaranteed total photogrammetric coverage of the entire lake; and the drone acquisitions were programmed in such a way as to allow the correct orthomosaic of the entire lake.

The first useful date that fulfilled all of these requirements was 5 April 2022. Among the products of the mission were a hundred RGB (red, green, and blue) and OCN (orange, ciano, and NIR) high-resolution data sets. Most of these data acquisitions were used to create an orthomosaic using the Agisoft Metashape software (Agisoft, St. Petersburg, Russia, 1.7.2), and this allowed a detailed observation of the entire surface of Lake Avernus. Additionally, the integrated multispectral data acquired by the drone were used to re-calibrate the satellite information for the purpose of monitoring (Figure 3), and a new NDTI index was applied over the time interval 2018–2022 (Figure 4).

### 4.3. In-Situ Sampling and In-Lab Taxonomy

A sample of the 2022 Lake Avernus bloom was collected in sterilized glass bottles on 29 March 2022, and kept refrigerated until the extraction. Main water parameters were measured at the sampling spot (LAT 40°50′25.43″ N LON 14°4′18.11″ E): pH (Violab) 7.78; temperature (air—Extech, water—TFA) 16 °C; and SAL (optical refractometer MR100ATC, AQL, Terlizzi (BA), Italy) 0 ‰. The presence and identity of the cyanobacterial community in the sample were first assessed by visual observation using a Motic Panthera C2 microscope (Motic, Barcelona, Spain). The cyanobacterial species composition was determined by 16S rRNA metagenomic analysis using a standardized and previously validated procedure [2]. 

Two mL of cyanobacterial cell suspension stored in RNA was later centrifuged for 10 min at 10,000 rpm; the supernatant was discarded, and the pellet was washed with 5 mL of PBS and the suspension centrifuged for 2 min at 10,000 rpm. Two mL of lysis buffer (200 mM Tris-Cl, 50 mM EDTA, 1.4 M NaCl, 2% CTAB, 0.5% PVP, all in milliQ^®^-H_2_O) was added to the pellet. After 3 min of sonication and the addition of 140 μL of lysozyme (20 mg/mL), the mixtures were vortexed and incubated at 37 °C for 1 h in a thermomixer at 1,400 rpm. After addition of 4 μL of β-mercaptoethanol, 100 μL of 10% SDS, 6 μL of RNase A (100 mg/mL), and 112 μL of proteinase K (10 mg/mL), the tube was incubated at 55 °C for another hour in a thermomixer (1400 rpm). The microcentrifuge tube was spun for 4 min at 10,000 rpm, and the clear middle phase was transferred to a new microcentrifuge tube containing 2 mL of CHCl_3_ and centrifuged for 10 min at 10,000 rpm. After two further CHCl_3_ washes, the supernatant was transferred to a new microcentrifuge tube containing 1 mL of 100% aqueous isopropyl alcohol and 10% (*v*/*v*) 3M NaOAc (pH 5.5) at room temperature. The precipitated DNA was left to precipitate overnight, then spun down at top speed for 30 min (4 °C), washed with ice-cold 70% ethanol, dried, and dissolved in ~60 μL of water. Amplification of 16S rRNA genes was performed by PCR using the universal primers 27F (5′-AGAGTTTGATCCTGGCTCAG-3′) and 1492R (5′-GGTTACCTTGTTACGACTT-3′) under the following conditions: initial denaturation at 95 °C for 45 s, followed by 33 cycles of 95 °C for 30 s, 48 °C for 30 s, and 72 °C for 90 s, with a final extension of 72 °C for 10 min. Amplification of cyanobacterial 16S rRNA genes was also performed by PCR using the specific primers CYA106F (5′-CGGACGGGTGAGTAACGCGTGA-3′) and CYA781R(a) (5′GACTACTGGGGTATCTAATCCCATT-3′) [31] under the following conditions: initial denaturation at 94 °C for 45 s, followed by 33 cycles of 94 °C for 1 min, 60 °C for 1 min, and 72 °C for 45 s, with a final extension 72 °C for 10 min. The reaction mixture (50 μL) contained: 27 μL of H_2_O, 2 μL of Mg^2+^, 3 μL of DMSO, 1.5 μL of dNTP (10 mM), 5 μL of Taq buffer (KAPA), 5 μL of forward primer (10 μM), 5 μL of reverse primer (10 μM), 1 μL of KAPA Taq DNA polymerase (5 U/μL), and 0.5 μL of DNA. The PCR products of the expected size (~1465 and ~670 bp) were purified from the agarose gel using the QIAquick gel ex kit (Qiagen, Germantown, MD, USA), and 16S rRNA sequences were analyzed using BLASTn (http://www.ncbi.nlm.nih.gov/, accessed 26 July 2022) [32]. Phylogenetic analyses (Figure S1) were performed using the MEGA 5.05 software package [33]. 

### 4.4. Metabolomic Profiling through HRMS/MS-Based Molecular Networking

The collected biomass (1L) was directly sonicated (Argo Lab, Carpi, Italy) for 15 min and extracted with MeOH (100%, 2.5 L), MeOH/CHCl_3_ (1:1, 2 L), and CHCl_3_ (100%, 1.5 L). The extracts were concentrated in vacuo, affording 8.5 g, 1.1 g, and 74 mg of the three extracts, respectively. The extracts were re-suspended in MeOH (100%) at a concentration of 5 mg/mL, filtered (Whatman, Cleves, OH, USA, 0.2 µm), and subjected to LC-HRMS and LC-HRMS/MS analyses using a Thermo LTQ Orbitrap XL high-resolution ESI mass spectrometer (Thermofisher, Waltham MA, USA) coupled to an Agilent model 1100 LC system. A 5-µm Kinetex C18 column (100 × 2.10 mm), maintained at room temperature, was eluted at 200 µL min^−1^ with H_2_O (supplemented with 0.1% HCOOH) and MeOH using a gradient elution. The gradient program was as follows: 10% MeOH for 3 min, 10–100% MeOH for 30 min, and 100% MeOH for 7 min. Mass spectra were acquired in positive ion detection mode. MS parameters utilized a spray voltage of 4.8 kV, a capillary temperature of 285 °C, a sheath gas rate of 32 units N_2_ (ca. 150 mL/min), and an auxiliary gas rate of 15 units N_2_ (*ca.* 50 mL/min). The MS method involved five HRMS/MS scans after each full MS scan for the five most intense ions detected in the spectrum (data-dependent acquisition mode, DDA). The *m*/*z* range for data-dependent acquisition was set between 150 and 2000 amu. HRMS/MS scans were obtained for selected ions with CID fragmentation using an isolation width of 2.0, a normalized collision energy of 35, an activation Q of 0.250, and an activation time of 30 ms. The data were analyzed using Thermo Xcalibur software (2.2 SP1 build 48). Raw files were imported into MZmine 2.53 [34]. The mass detection was performed on raw data and exact masses with mass level 1 and centroided masses with mass level 2, by keeping the noise level at 10,000. Chromatograms were built using an ADAP module with a minimum height of 10,000 and a *m*/*z* tolerance of 0.01 (or 20 ppm). For the chromatogram deconvolution, the local minimum search algorithm was used with the following settings: chromatographic threshold = 5%, minimum retention time range = 0.20 min, minimum relative height = 30%, minimum absolute height = 10,000, minimum ratio of the peak top/edge = 1.3, and peak duration range = 0.0–6.0 min. Peak alignment was performed using the Join aligner algorithm (*m*/*z* tolerance at 0.005 (or 10 ppm), absolute RT tolerance at 0.3 min). [M + Na–H], [M + K–H], [M + Mg–2H], [M + NH_3_], [M-Na + NH_4_], [M + 1, ^13^C], [M–^35^Cl + ^37^Cl], and [M + ^56^Fe–3H] adducts were filtered out by setting the maximum relative height at 100%. Peaks without associated MS/MS spectra were finally filtered out of the peak list. Clustered data were then exported to an mgf file for GNPS, while chromatographic data, including retention times, peak areas, and peak heights, were exported to a csv file. A feature-based molecular network [29] was generated on GNPS’s online platform [35], with the following parameters: the precursor ion mass tolerance was set to 0.02 Da, the MS/MS fragment ion tolerance to 0.02 Da, a cosine score above 0.6, and more than five matched peaks. For GNPS’s library search, a cosine score of 0.6 and at least six matched peaks were set. The DEREPLICATOR was used to annotate MS/MS spectra [18]. The molecular network was visualized using Cytoscape software (3.9.1) [36].

Purification of anabaenopeptin B (**2**) from an aliquot of the MeOH extract (300 mg) was achieved through HPLC (Agilent 1260 Infinity Quaternary LC apparatus (Agilent Technology, Cernusco sul Naviglio, Italy, equipped with a diode-array detector, DAD) on a Luna C18 column (250 × 10 mm, 10 µm) (Phenomenex, Torrance, CA, USA). [Eluent A: 0.1% HCOOH in H_2_O; eluent B: MeOH; gradient program: 10% B over 3 min, 10% → 100% B over 30 min, 100% B over 7 min; flow rate 5 mL min^−1^, wavelength 280 nm], affording 11.5 mg of anabenopeptin B (**2**, *t*_R_ = 20 min). 

*Anabaenopeptin B* (**2**): white amorphous solid, HRESIMS (positive ion mode, MeOH) *m*/*z* 837.4618 [M + H]^+^ (C_41_H_61_N_10_O_9,_ calcd. 837.4617). HRMS/MS spectrum: Figure S4; ^1^H, ^13^C HSQC NMR (DMSO-d_6_): Figures S17–S19 and Table S1.

## Figures and Tables

**Table 1:** High-resolution MS/MS fragmentations of anabaenopeptin variants found in the Lake Avernus 2022 bloom. Fragment a_1_ originates from the loss of a single amino acid residue (aa-1); fragment a_34_ is due to the loss of two amino acids (aa-3 and aa-4); and fragment a_134_ is due to the loss of three residues (aa-1, aa-3, and aa-4) (see Figure 8). This code is applied to all the fragment ions. *n.o.* = not observed.

Fragment ion	AP-A (1)	AP-B (2)	Osc-B (3)	Osc-Y (4)	AP-F (5)	AP-820 (6)	AP-679 (7)	AP-AV875 (8)	AP-AV871 (9)	AP-AV834 (10)	AP-AV829 (11)	AP-AV827 (12)	AP-AV822 (13)	AP-AV693 (14)
[M + H]^+^	844.4233 C_44_H_58_N_7_O_10_^+^	837.4611 C_41_H_61_N_10_O_9_^+^	869.4333 C_41_H_61_N_10_O_9_S^+^	858.4384 C_45_H_60_N_7_O_10_^+^	851.4767 C_42_H_63_N_10_O_9_^+^	821.4668 C_41_H_61_N_10_O_8_^+^	680.3758 C_35_H_50_N_7_O_7_^+^	876.3956 C_44_H_58_N_7_O_10_S^+^	872.4543 C_46_H_62_N_7_O_10_^+^	835.4822 C_42_H_63_N_10_O_8_^+^	830.4075 C_43_H_56_N_7_O_10_^+^	828.4288 C_44_H_58_N_7_O_9_^+^	823.4456 C_40_H_59_N_10_O_9_^+^	694.4003 C_36_H_52_N_7_O_7_^+^
[M + 2H]^2+^	*n.o.*	419.2344 C_41_H_62_N_10_O_9_^2+^	435.2200 C_41_H_62_N_10_O_9_S^2+^	*n.o.*	426.2420 C_42_H_64_N_10_O_9_^2+^	411.2368 C_41_H_62_N_10_O_8_^2+^	*n.o.*	*n.o.*	*n.o.*	418.2443 C_42_H_64_N_10_O_8_^2+^	*n.o.*	*n.o.*	412.2288 C_40_H_60_N_10_O_9_^2+^	*n.o.*
a_1_	663.3494 C_35_H_47_N_6_O_7_^+^	663.3496 C_35_H_47_N_6_O_7_^+^	695.3209 C_35_H_47_N_6_O_7_S^+^	677.3651 C_36_H_49_N_6_O_7_^+^	*n.o.*	647.3547 C_35_H_47_N_6_O_6_^+^	663.3520 C_35_H_47_N_6_O_7_^+^	695.3210 C_35_H_47_N_6_O_7_S^+^	677.3644 C_36_H_49_N_6_O_7_^+^	*n.o.*	649.3358 C_34_H_45_N_6_O_7_^+^	647.3565 C_35_H_47_N_6_O_6_^+^	649.3361 C_35_H_47_N_6_O_7_^+^	677.3678 C_36_H_49_N_6_O_7_^+^
a_1_-CO	637.3700 C_34_H_49_N_6_O_6_^+^	637.3700 C_34_H_49_N_6_O_6_^+^	669.3414 C_34_H_49_N_6_O_6_S^+^	651.3860 C_35_H_51_N_6_O_6_^+^	651.3856 C_35_H_51_N_6_O_6_^+^	621.3739 C_34_H_49_N_6_O_5_^+^	637.3727 C_34_H_49_N_6_O_6_^+^	669.3417 C_34_H_49_N_6_O_6_S^+^	651.3853 C_35_H_51_N_6_O_6_^+^	635.3898 C_35_H_51_N_6_O_5_^+^	623.3562 C_33_H_47_N_6_O_6_^+^	621.3772 C_34_H_49_N_6_O_5_^+^	623.3562 C_33_H_47_N_6_O_6_^+^	651.3883 C_35_H_51_N_6_O_6_^+^
a_2_	509.2753 C_28_H_37_N_4_O_5_^+^	509.2753 C_28_H_37_N_4_O_5_^+^	*n.o.*	523.2907 C_29_H_39_N_4_O_5_^+^	523.2905 C_29_H_39_N_4_O_5_^+^	493.2794 C_28_H_37_N_4_O_4_^+^	509.2773 C_28_H_37_N_4_O_5_^+^	541.2465 C_28_H_37_N_4_O_5_S^+^	523.2903 C_29_H_39_N_4_O_5_^+^	*n.o.*	495.2606 C_27_H_35_N_4_O_5_^+^	493.2817 C_28_H_37_N_4_O_4_^+^	495.2608 C_27_H_35_N_4_O_5_^+^	523.2933 C_29_H_39_N_4_O_5_^+^
a_3_	745.3551 C_39_H_49_N_6_O_9_^+^	*n.o.*	*n.o.*	745.3556 C_39_H_49_N_6_O_9_^+^	*n.o.*	*n.o.*	*n.o.*	*n.o.*	759.3680 C_40_H_51_N_6_O_9_^+^	*n.o.*	731.3419 C_38_H_47_N_6_O_9_^+^	*n.o.*	724.3792 C_35_H_50_N_9_O_8_^+^	*n.o.*
a_4_	667.3443 C_34_H_47_N_6_O_8_^+^	660.3827 C_31_H_50_N_9_O_7_^+^	692.3532 C_31_H_50_N_9_O_7_S^+^	681.3602 C_35_H_49_N_6_O_8_^+^	674.3980 C_32_H_52_N_9_O_7_^+^	*n.o.*	503.2991 C_25_H_39_N_6_O_5_^+^	699.3160 C_34_H_47_N_6_O_8_S^+^	695.3751 C_36_H_51_N_6_O_8_^+^	674.3971 C_32_H_52_N_9_O_7_^+^	*n.o*	667.3468 C_34_H_47_N_6_O_8_^+^	646.3658 C_30_H_48_N_9_O_7_^+^	517.3123 C_26_H_41_N_6_O_5_^+^
a_5_	759.3706 C_40_H_51_N_6_O_9_^+^	752.4084 C_37_H_54_N_9_O_8_^+^	784.3798 C_37_H_54_N_9_O_8_S^+^	773.3864 C_41_H_53_N_6_O_9_^+^	766.4242 C_38_H_56_N_9_O_8_^+^	736.4126 C_37_H_54_N_9_O_7_^+^	*n.o.*	791.3450 C_40_H_51_N_6_O_9_S^+^	787.4025 C_42_H_55_N_6_O_9_^+^	750.4283 C_38_H_56_N_9_O_7_^+^	759.3699 C_40_H_51_N_6_O_9_^+^	743.3790 C_40_H_51_N_6_O_8_^+^	*n.o.*	*n.o.*
a_6_-H_2_O	679.3445 C_35_H_47_N_6_O_8_^+^	672.3821 C_32_H_50_N_9_O_7_^+^	*n.o.*	693.3613 C_36_H_49_N_6_O_8_^+^	686.3981 C_33_H_52_N_9_O_7_^+^	*n.o.*	*n.o.*	*n.o.*	*n.o.*	*n.o.*	*n.o.*	663.3494 C_35_H_47_N_6_O_7_^+^	*n.o.*	*n.o.*
a_14_-CO	460.2912 C_24_H_38_N_5_O_4_^+^	460.2914 C_24_H_38_N_5_O_4_^+^	492.2621 C_24_H_38_N_5_O_4_S^+^	474.3068 C_25_H_40_N_5_O_4_^+^	474.3068 C_25_H_40_N_5_O_4_^+^	460.2908 C_24_H_38_N_5_O_4_^+^	*n.o.*	*n.o.*	474.3057 C_25_H_40_N_5_O_4_^+^	*n.o.*	*n.o.*	*n.o.*	446.2777 C_23_H_36_N_5_O_4_^+^	*n.o.*
a_34_	568.2759 C_29_H_38_N_5_O_7_^+^	561.3142 C_26_H_41_N_8_O_6_^+^	561.3128 C_26_H_41_N_8_O_6_^+^	568.2761 C_29_H_38_N_5_O_7_^+^	561.3140 C_26_H_41_N_8_O_6_^+^	*n.o.*	404.2306 C_20_H_30_N_5_O_4_^+^	568.2762 C_29_H_38_N_5_O_7_^+^	582.2911 C_30_H_40_N_5_O_7_^+^	*n.o.*	554.2619 C_28_H_36_N_5_O_7_^+^	568.2776 C_29_H_38_N_5_O_7_^+^	547.2983 C_25_H_39_N_8_O_6_^+^	404.2308 C_20_H_30_N_5_O_4_^+^
a_34_-H_2_O	550.2654 C_29_H_36_N_5_O_6_^+^	543.3035 C_26_H_39_N_8_O_5_^+^	543.3025 C_26_H_39_N_8_O_5_^+^	550.2656 C_29_H_36_N_5_O_6_^+^	543.3033 C_26_H_39_N_8_O_5_^+^	543.3024 C_26_H_39_N_8_O_5_^+^	*n.o.*	550.2650 C_29_H_36_N_5_O_6_^+^	*n.o.*	543.3033 C_26_H_39_N_8_O_5_^+^	536.2494 C_28_H_34_N_5_O_6_^+^	550.2672 C_29_H_36_N_5_O_6_^+^	529.2898 C_25_H_37_N_8_O_5_^+^	*n.o.*
a_45_	582.2916 C_30_H_40_N_5_O_7_^+^	575.3297 C_27_H_43_N_8_O_6_^+^	607.3010 C_27_H_43_N_8_O_6_S^+^	596.3074 C_31_H_42_N_5_O_7_^+^	589.3452 C_28_H_45_N_8_O_6_^+^	575.3285 C_27_H_43_N_8_O_6_^+^	418.2458 C_21_H_32_N_5_O_4_^+^	*n.o.*	610.3218 C_32_H_44_N_5_O_7_^+^	589.3442 C_28_H_45_N_8_O_6_^+^	582.2936 C_30_H_40_N_5_O_7_^+^	582.2925 C_30_H_40_N_5_O_7_^+^	575.3289 C_27_H_43_N_8_O_6_^+^	432.2615 C_22_H_34_N_5_O_4_^+^
a_134_ + H_2_O	379.2337 C_19_H_31_N_4_O_4_^+^	379.2346 C_19_H_31_N_4_O_4_^+^	*n.o.*	379.2338 C_19_H_31_N_4_O_4_^+^	379.2337 C_19_H_31_N_4_O_4_^+^	*n.o.*	*n.o.*	379.2341 C_19_H_31_N_4_O_4_^+^	*n.o.*	*n.o.*	*n.o.*	379.2348 C_19_H_31_N_4_O_4_^+^	*n.o.*	*n.o.*
a_134_ + H_2_O + CO	405.2129 C_20_H_29_N_4_O_5_^+^	*n.o.*	*n.o.*	405.2130 C_20_H_29_N_4_O_5_^+^	*n.o.*	*n.o.*	*n.o.*	405.2132 C_20_H_29_N_4_O_5_^+^	*n.o.*	*n.o.*	391.1985 C_19_H_27_N_4_O_5_^+^	405.2143 C_20_H_29_N_4_O_5_^+^	*n.o.*	*n.o.*
a_126_	362.2070 C_19_H_28_N_3_O_4_^+^	362.2073 C_19_H_28_N_3_O_4_^+^	394.1789 C_19_H_28_N_3_O_4_S^+^	376.2229 C_20_H_30_N_3_O_4_^+^	376.2227 C_20_H_30_N_3_O_4_^+^	346.2117 C_19_H_28_N_3_O_3_^+^	362.2085 C_19_H_28_N_3_O_4_^+^	*n.o.*	376.2224 C_20_H_30_N_3_O_4_^+^	360.2271 C_20_H_30_N_3_O_3_^+^	348.1920 C_18_H_26_N_3_O_4_^+^	*n.o.*	348.1926 C_18_H_26_N_3_O_4_^+^	376.2238 C_20_H_30_N_3_O_4_^+^
a_156_	*n.o.*	405.2495 C_21_H_33_N_4_O_4_^+^	*n.o.*	*n.o.*	419.2648 C_22_H_33_N_4_O_3_^+^	*n.o.*	*n.o.*	*n.o.*	*n.o.*	*n.o.*	*n.o.*	*n.o.*	*n.o.*	*n.o.*
a_156_-H_2_O	387.2387 C_21_H_31_N_4_O_3_^+^	387.2386 C_21_H_31_N_4_O_3_^+^	*n.o.*	401.2539 C_22_H_35_N_4_O_4_^+^	401.2541 C_22_H_35_N_4_O_4_^+^	*n.o.*	*n.o.*	*n.o.*	401.2542 C_22_H_35_N_4_O_4_^+^	*n.o.*	*n.o.*	*n.o.*	387.2394 C_21_H_31_N_4_O_3_^+^	*n.o.*

## Data Availability

Data are contained within the article and Supplementary Materials.

## Supplementary Materials

The following supporting information can be downloaded at: https://www.mdpi.com/article/10.3390/toxins15120698/s1. Figure S1: 16S rRNA phylogenetic tree displaying the taxonomy of the cyanobacterial community inhabiting the 2022 Lake Avernus bloom. Figure S2: LC-HRMS total ion chromatogram (TIC) of the 2022 Lake Avernus bloom MeOH extract. Figure S3: HRMS/MS spectrum of AP-A (**1**) (*m*/*z* 844.4234). Figure S4: HRMS/MS spectrum of AP-B (**2**) (*m*/*z* 837.4618). Figure S5: HRMS/MS spectrum of Osc-B (**3**) (*m*/*z* 869.4333). Figure S6: HRMS/MS spectrum of Osc-Y (**4**) (*m*/*z* 858.4393). Figure S7: HRMS/MS spectrum of AP-F (**5**) (*m*/*z* 851.4768). Figure S8: HRMS/MS spectrum of AP-820 (**6**) (*m*/*z* 821.4668). Figure S9: HRMS/MS spectrum of AP-679 (**7**) (*m*/*z* 680.3764). Figure S10: HRMS/MS spectrum of AP-AV875 (**8**) (*m*/*z* 876.3962). Figure S11: HRMS/MS spectrum of AP-AV871 (**9**) (*m*/*z* 872.4542). Figure S12: HRMS/MS spectrum of AP-AV834 (**10**) (*m*/*z* 835.4816). Figure S13: HRMS/MS spectrum of AP-AV829 (**11**) (*m*/*z* 830.4081). Figure S14: HRMS/MS spectrum of AP-AV827 (**12**) (*m*/*z* 828.4289). Figure S15: HRMS/MS spectrum of AP-AV822 (**13**) (*m*/*z* 823.4464). Figure S16: HRMS/MS spectrum of AP-AV693 (**14**) (*m*/*z* 694.4003). Figure S17: ^1^H-NMR spectrum of AP-B (**2**) (700 MHz, DMSO-d_6_). Figure S18: ^13^C-NMR spectrum of AP-B (**2**) (700 MHz, DMSO-d_6_). Figure S19: HSQC NMR spectrum of AP-B (**2**) (700 MHz, DMSO-d_6_). Table S1: NMR data of AP-B (**2**) (700 MHz, DMSO-d_6_).
