# Supplementary material for: Lake Avernus Has Turned Red: Bioindicator Monitoring Unveils the Secrets of “Gates of Hades”
Source: Toxins (Basel). 2023 Dec 13;15(12):698. doi: 10.3390/toxins15120698 (PMC10747548; doi:10.3390/toxins15120698)
Supplement: Supplementary file 1 [file toxins-15-00698-s001.zip › toxins-2751995-supplementary.pdf]

toxins

MDPI

Supplementary Material

Lake Avernus Has Turned Red: Bioindicator Monitoring Unveils the Secrets of "Gates of Hades"

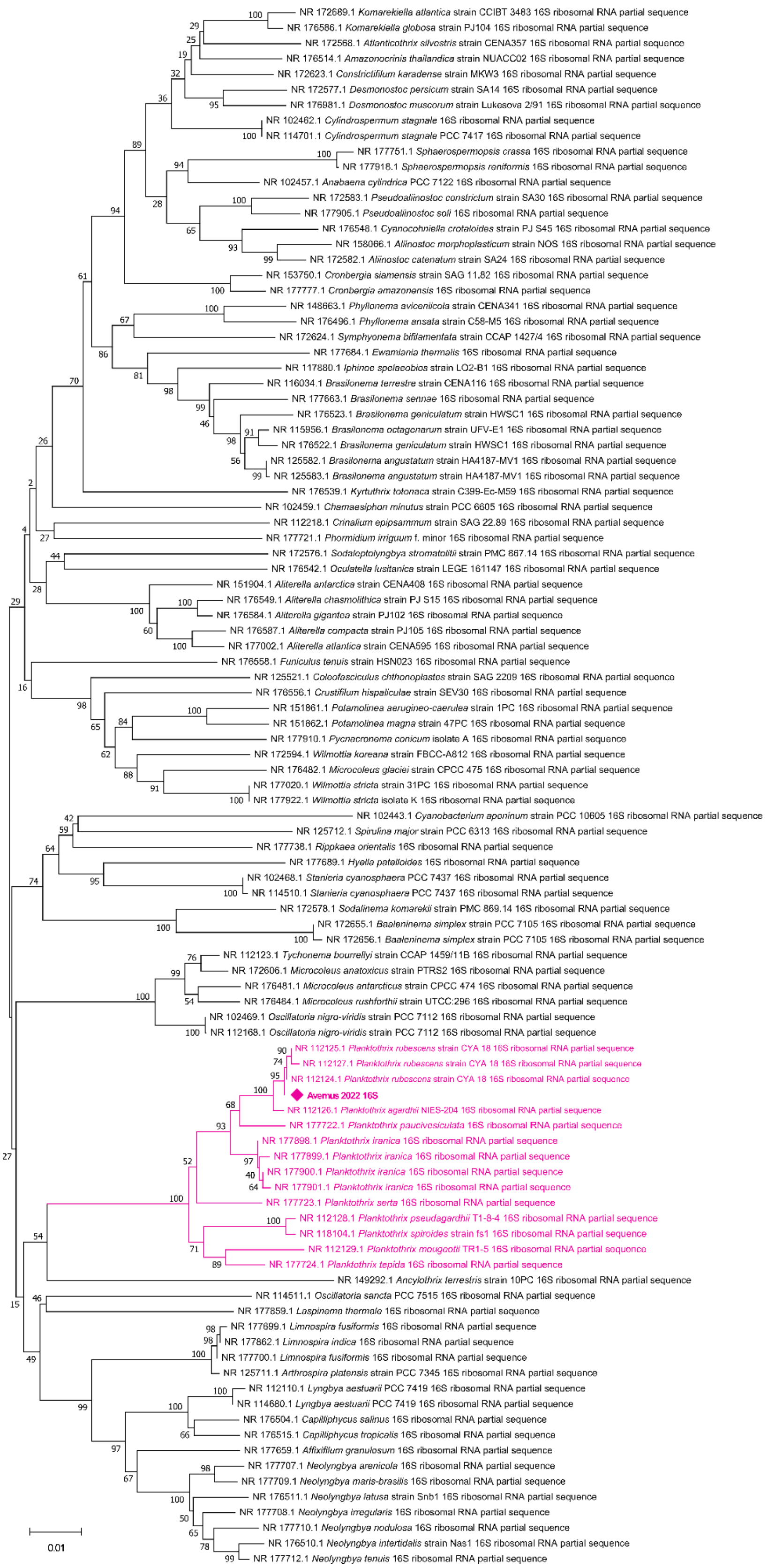

Figure S1. 16S rRNA gene neighbor-joining phylogenetic tree displaying the taxonomy of the cyanobacterial community inhabiting 2022 Lake Avernus bloom labeled with diamond (♦). Bootstrap values are given at nodes. Scale-bar represents the phylogenetic distance related to the number of nucleotide substitutions per site.

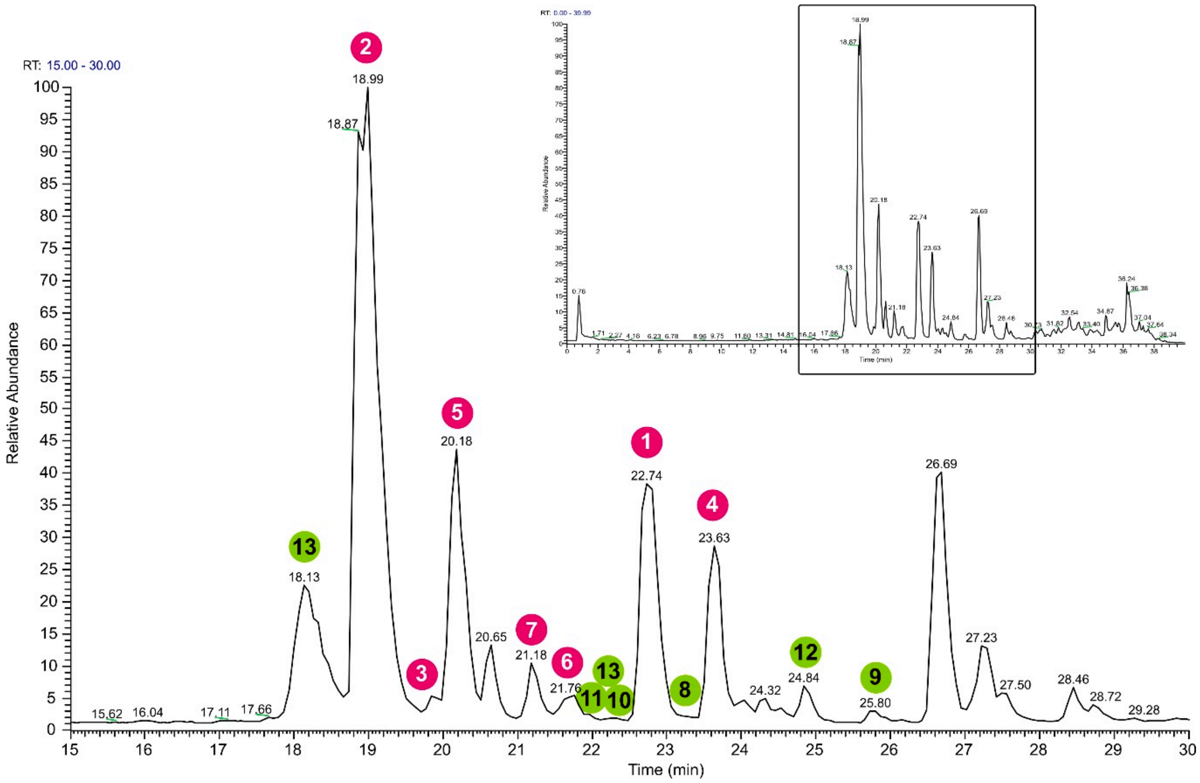

Figure S2. LC-HRMS total ion chromatogram (TIC) of the 2022 Lake Avernus bloom MeOH extract. Peak numbers refers to known (purple) and new (green) anabaenopeptin variants.

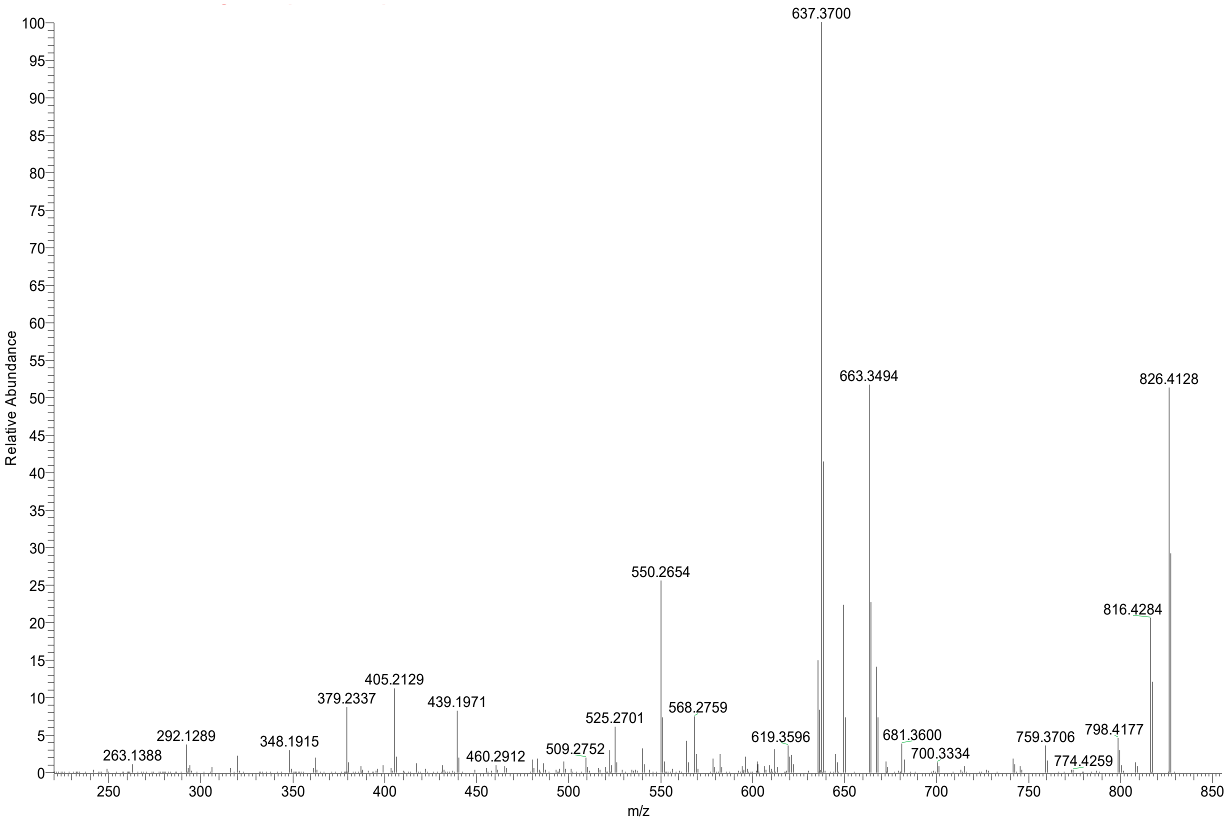

Figure S3. HRMS/MS spectrum of AP-A (**1**) (*m/z* 844.4234).

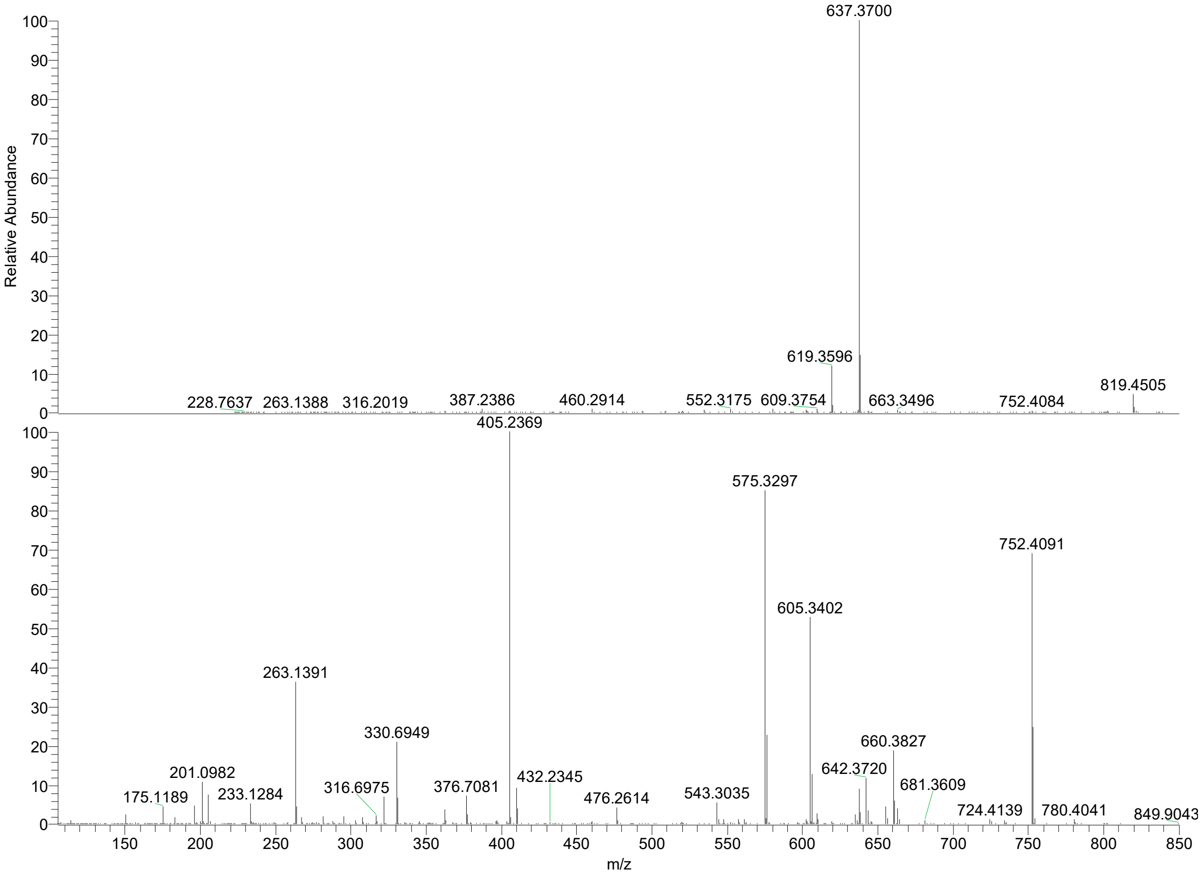

Figure S4. HRMS/MS spectrum of AP-B (**2**) (*m/z* 837.4618).

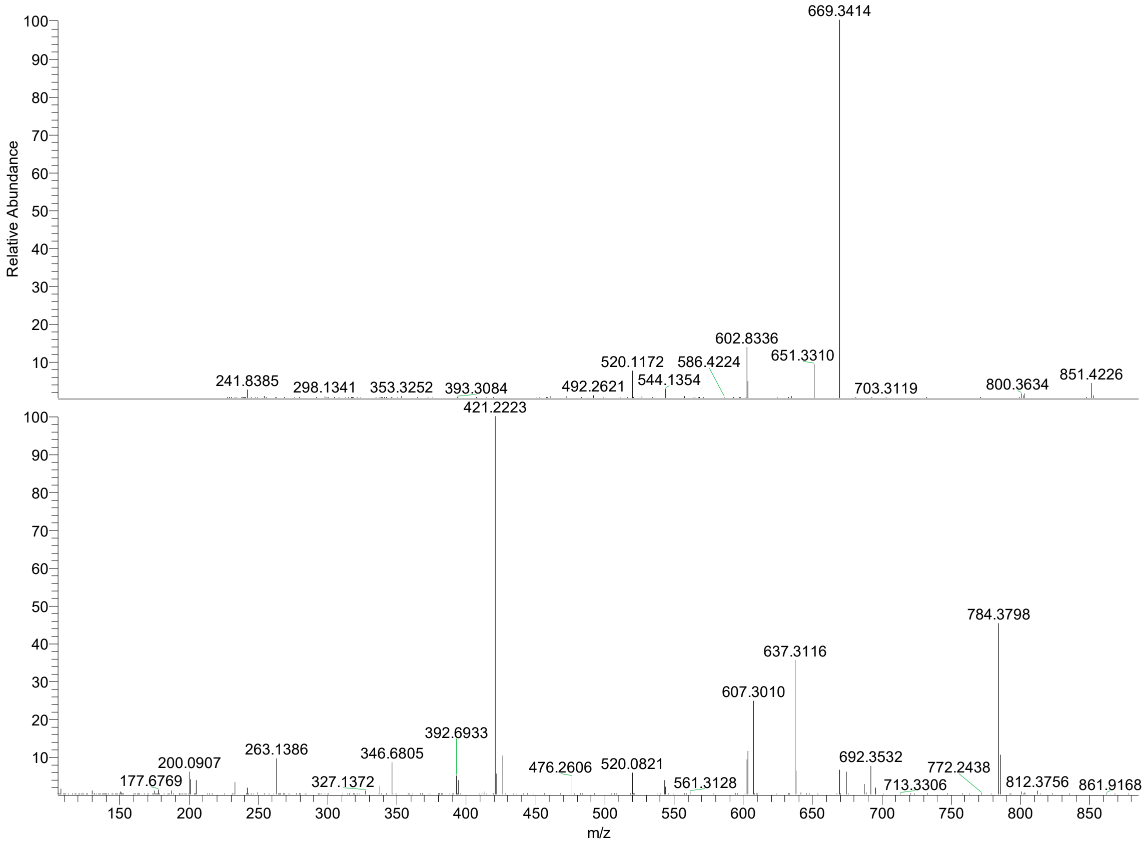

Figure S5. HRMS/MS spectrum of Osc-B (**3**) (*m/z* 869.4333).

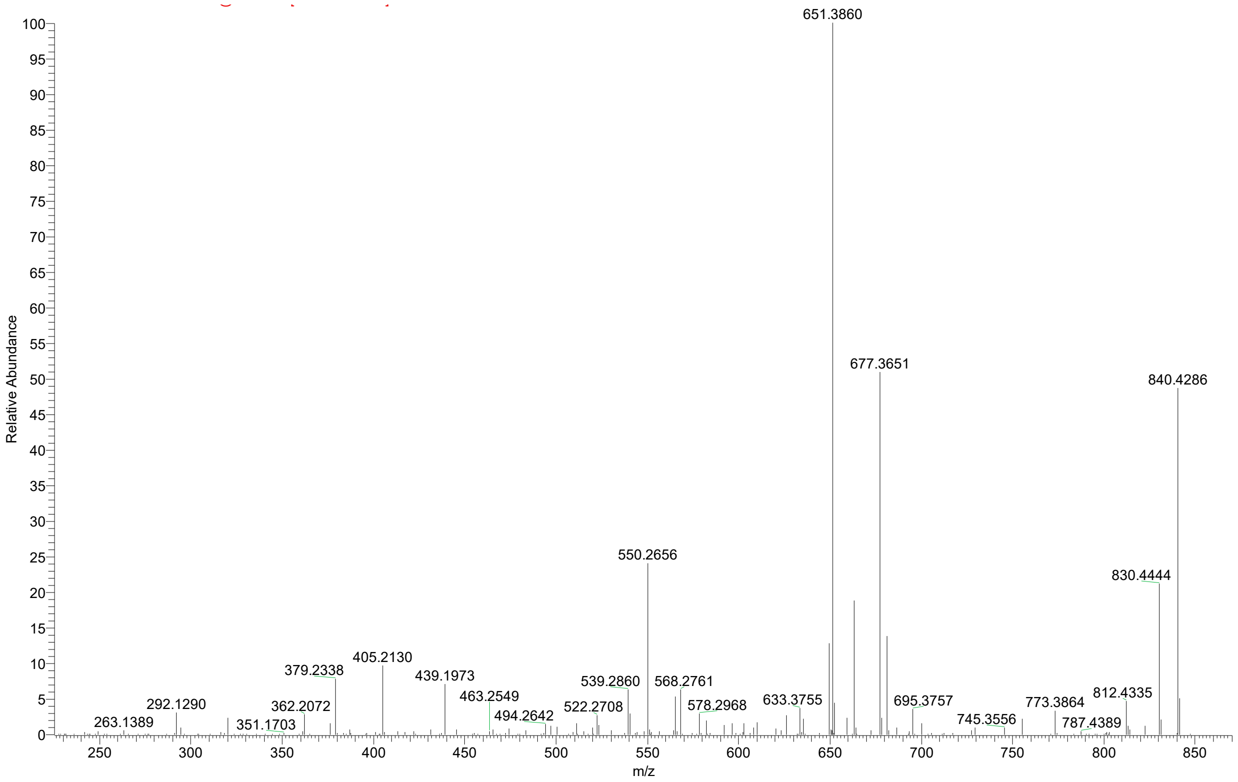

Figure S6. HRMS/MS spectrum of Osc-Y (**4**) (*m/z* 858.4393).

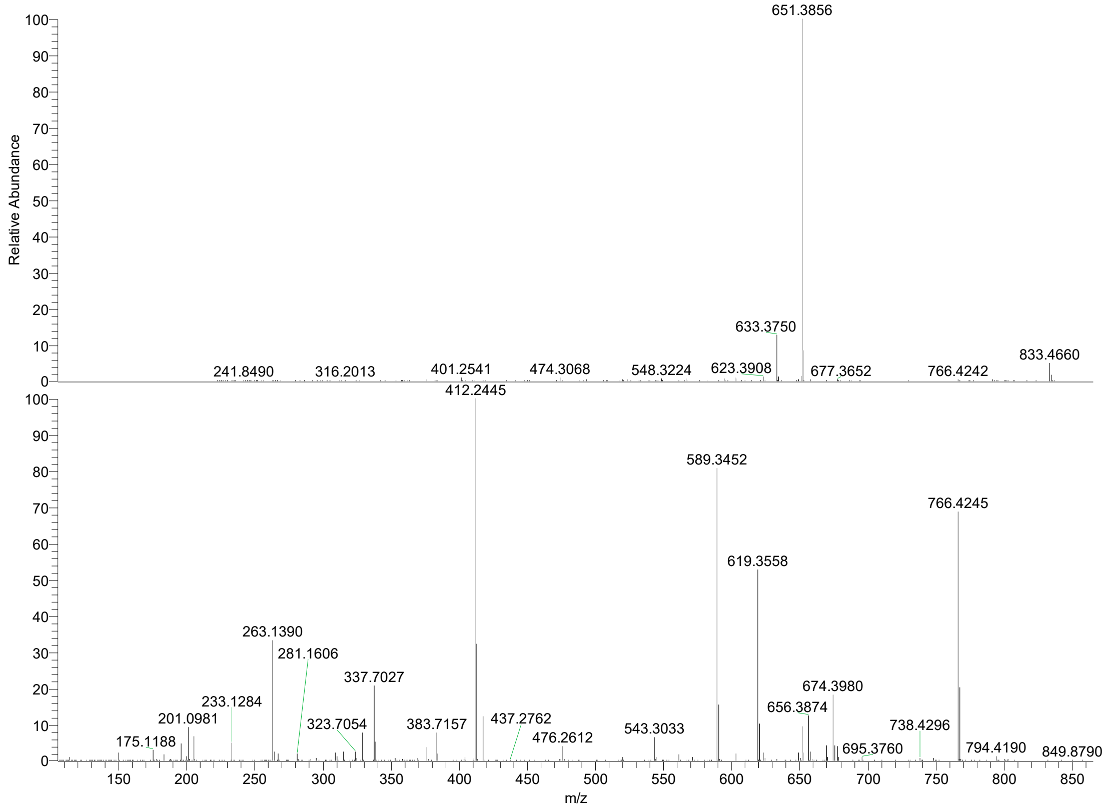

Figure S7. HRMS/MS spectrum of AP-F (**5**) (*m*/*z* 851.4768).

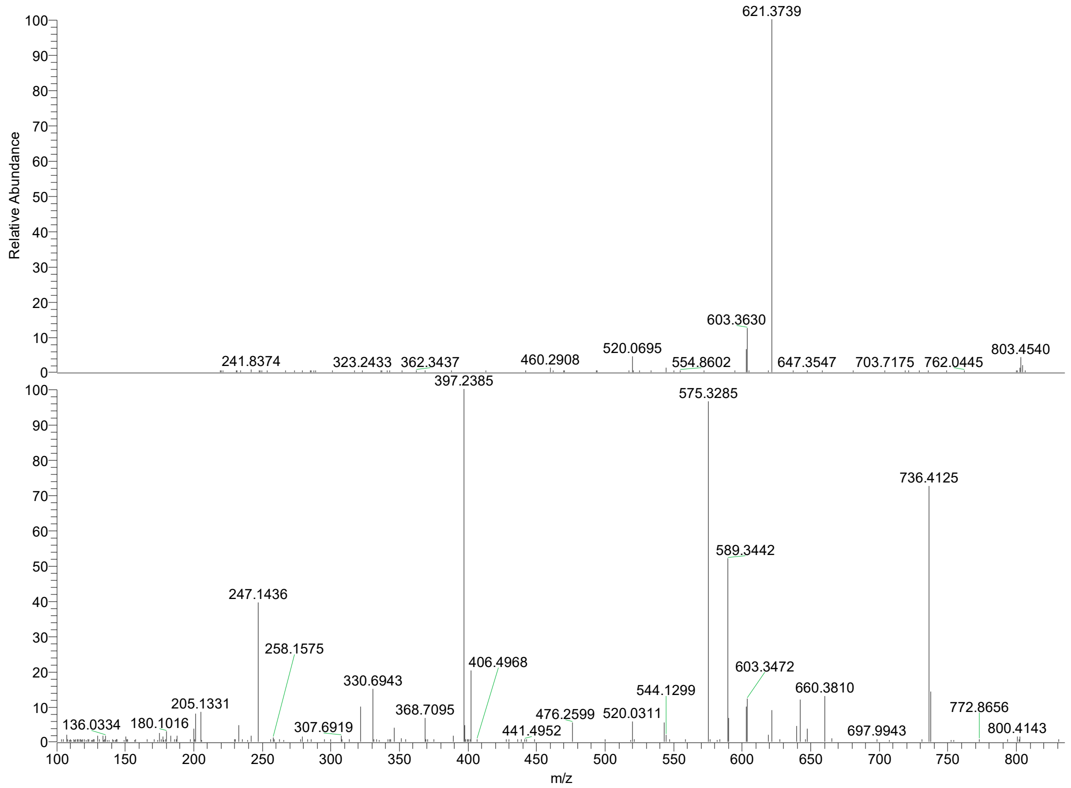

Figure S8. HRMS/MS spectrum of AP-820 (**6**) (*m*/*z* 821.4668).

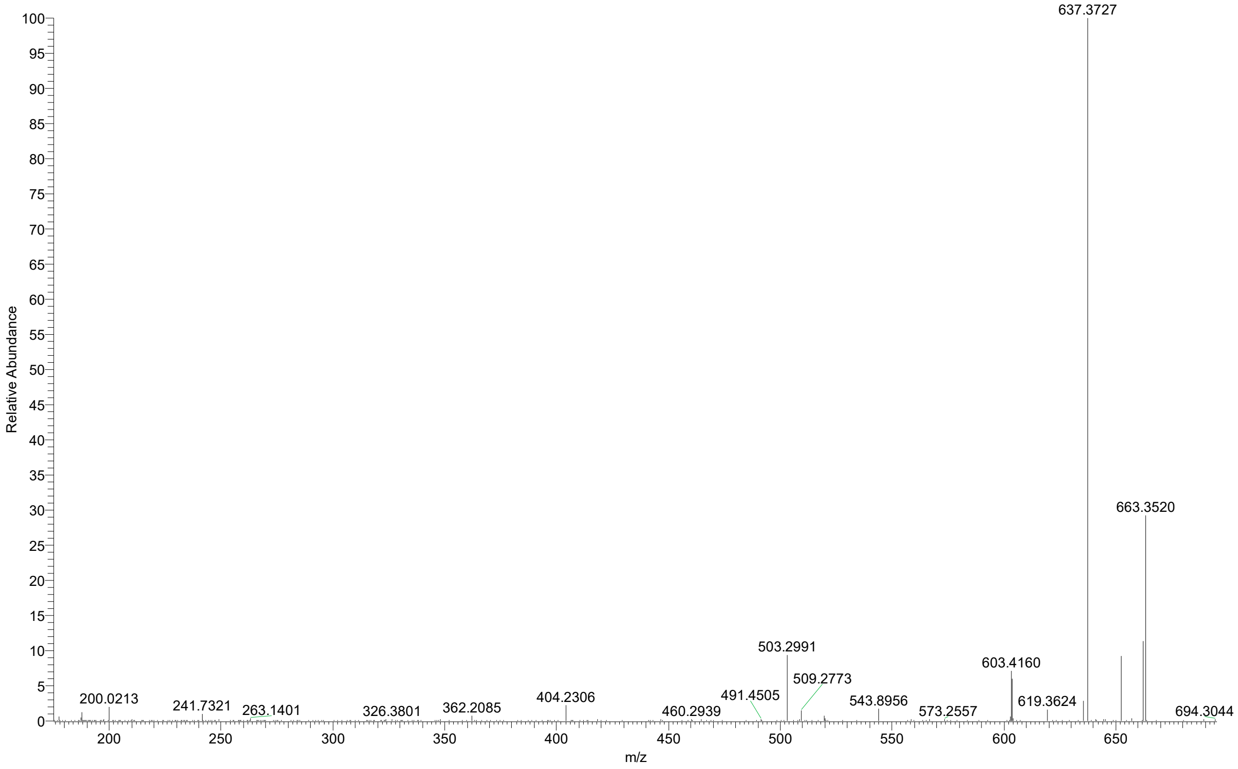

Figure S9. HRMS/MS spectrum of AP-679 (**7**) (*m/z* 680.3764).

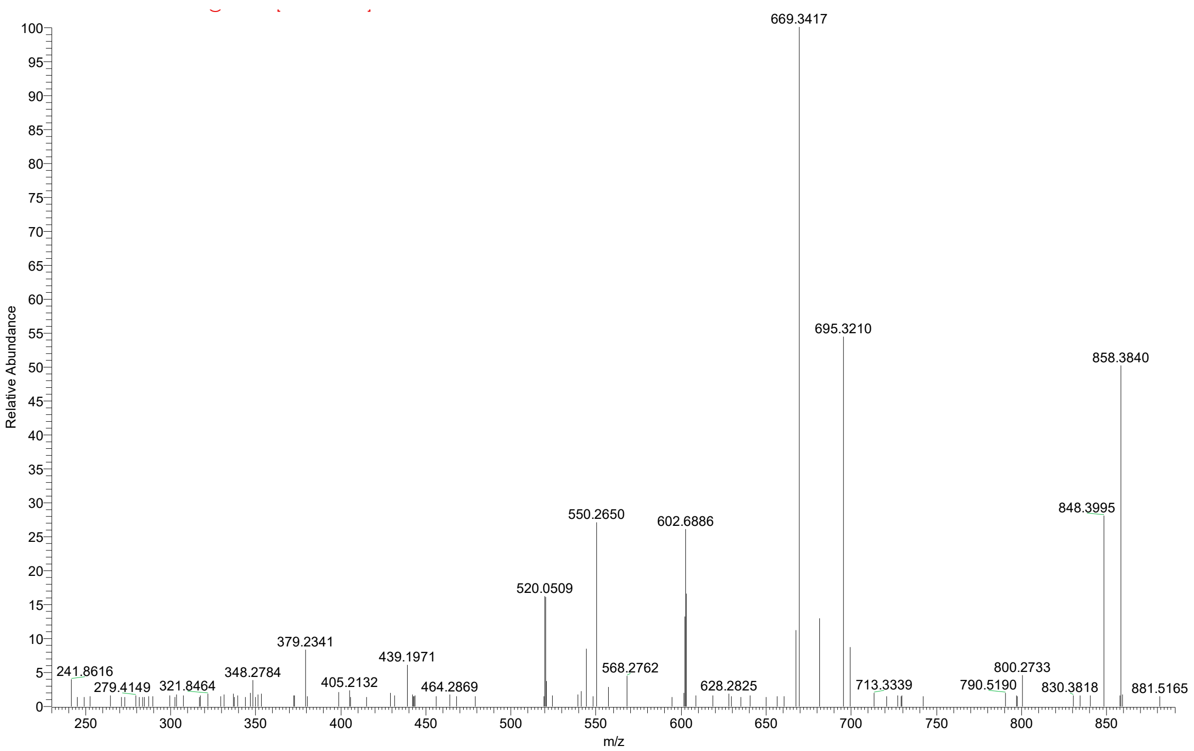

Figure S10. HRMS/MS spectrum of AP-AV875 (**8**) (*m/z* 876.3962).

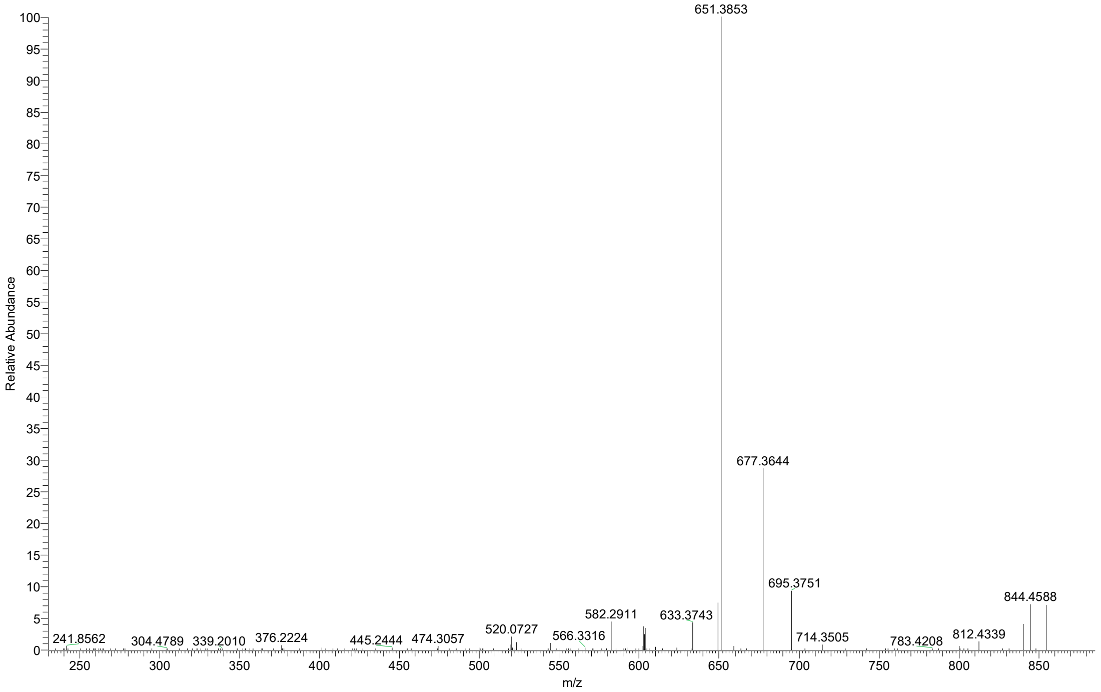

Figure S11. HRMS/MS spectrum of AP-AV871 (**9**) (*m/z* 872.4542).

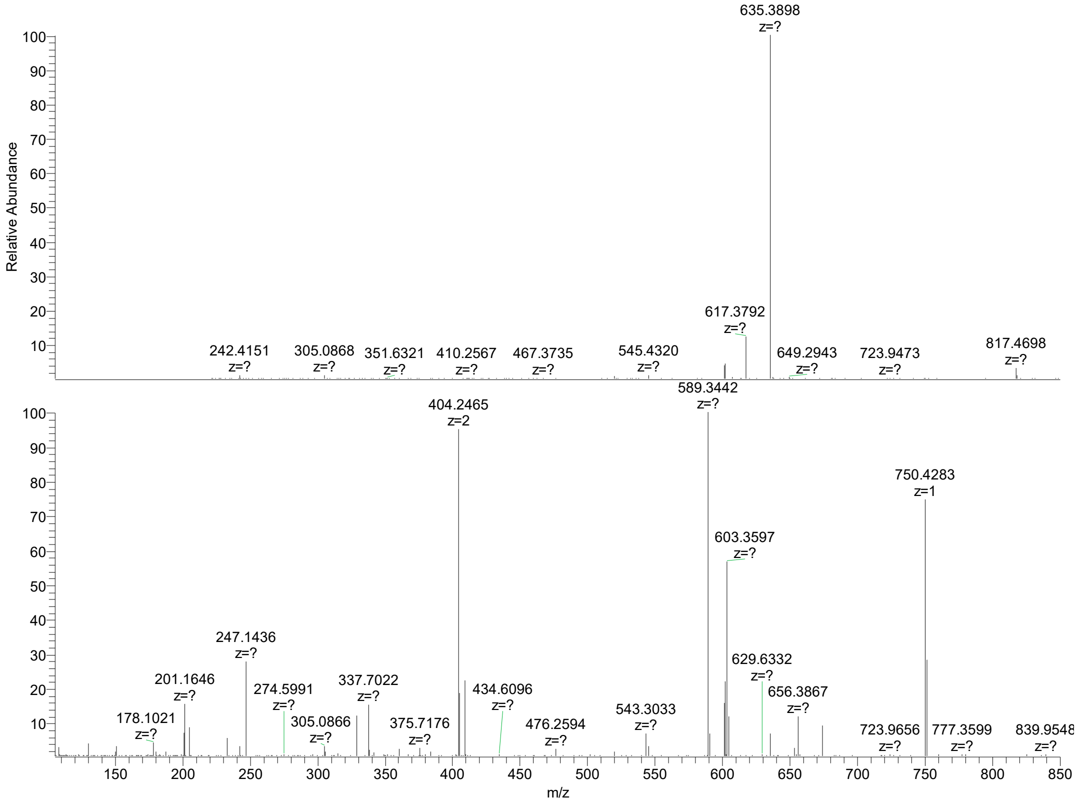

Figure S12. HRMS/MS spectrum of AP-AV834 (**10**) (*m/z* 835.4816).

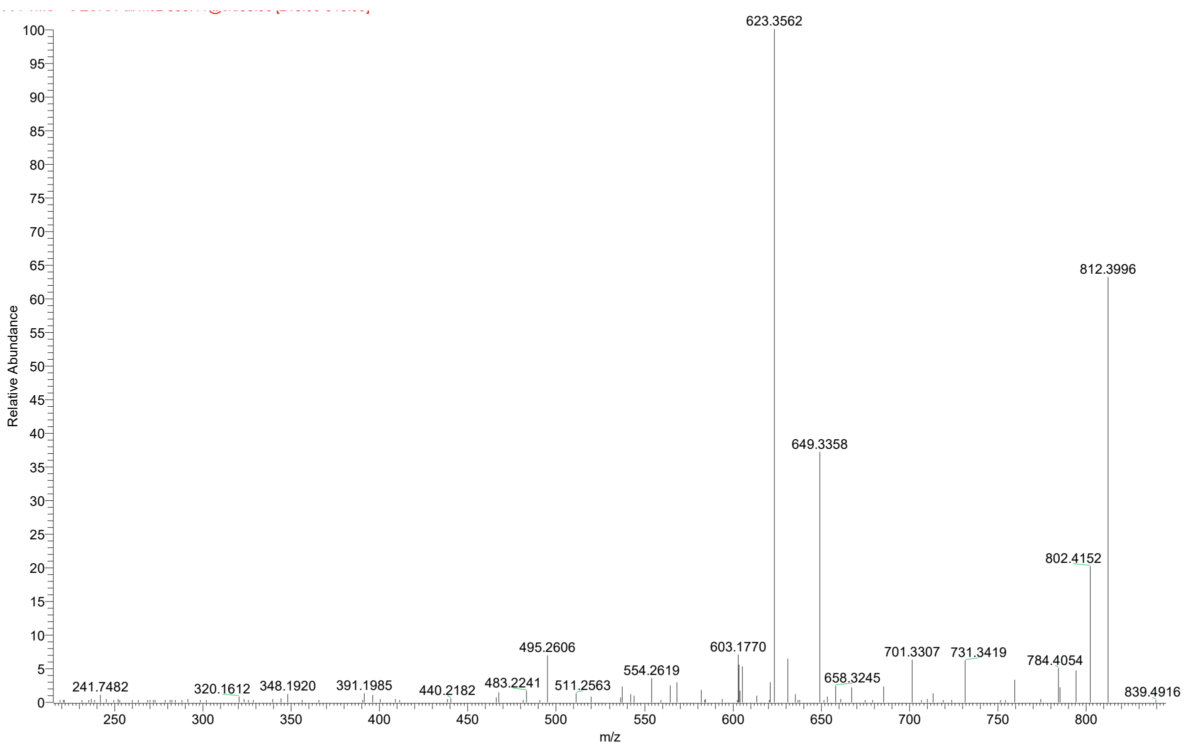

Figure S13. HRMS/MS spectrum of AP-AV829 (**11**) (*m*/*z* 830.4081).

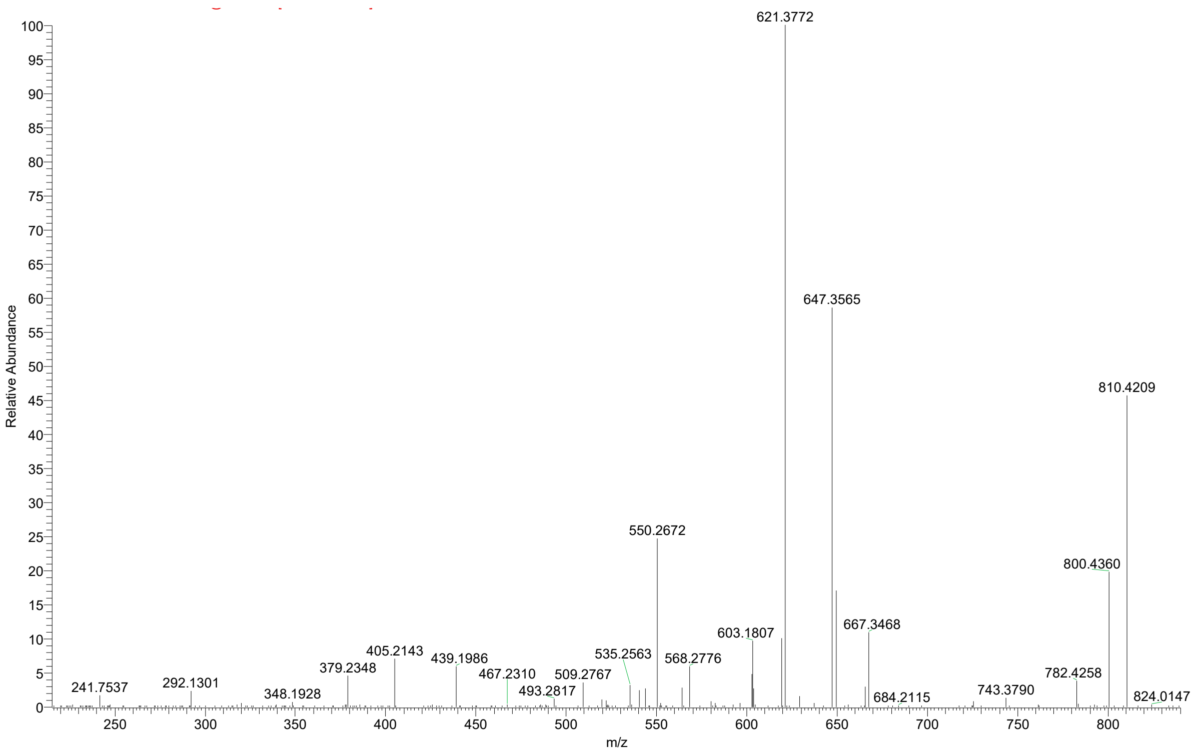

Figure S14. HRMS/MS spectrum of AP-AV827 (**12**) (*m*/*z* 828.4289).

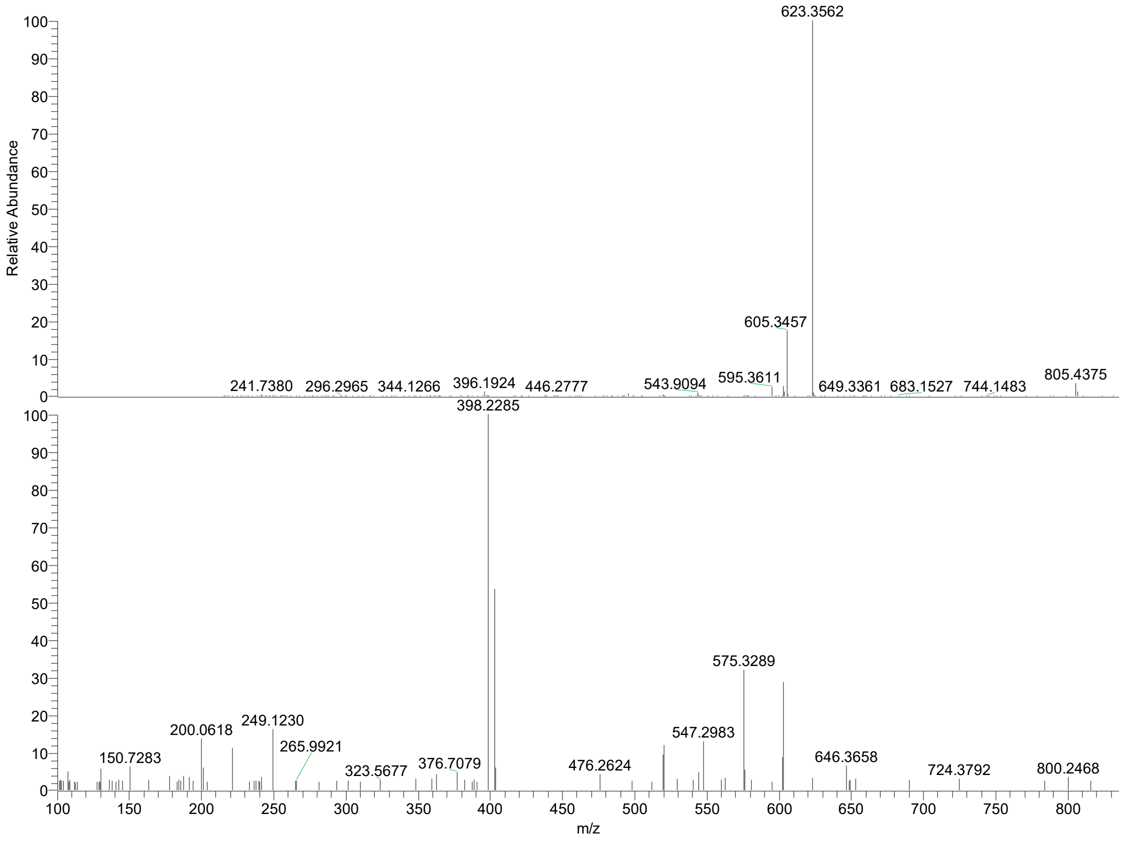

Figure S15. HRMS/MS spectrum of AP-AV822 (**13**) (*m/z* 823.4464).

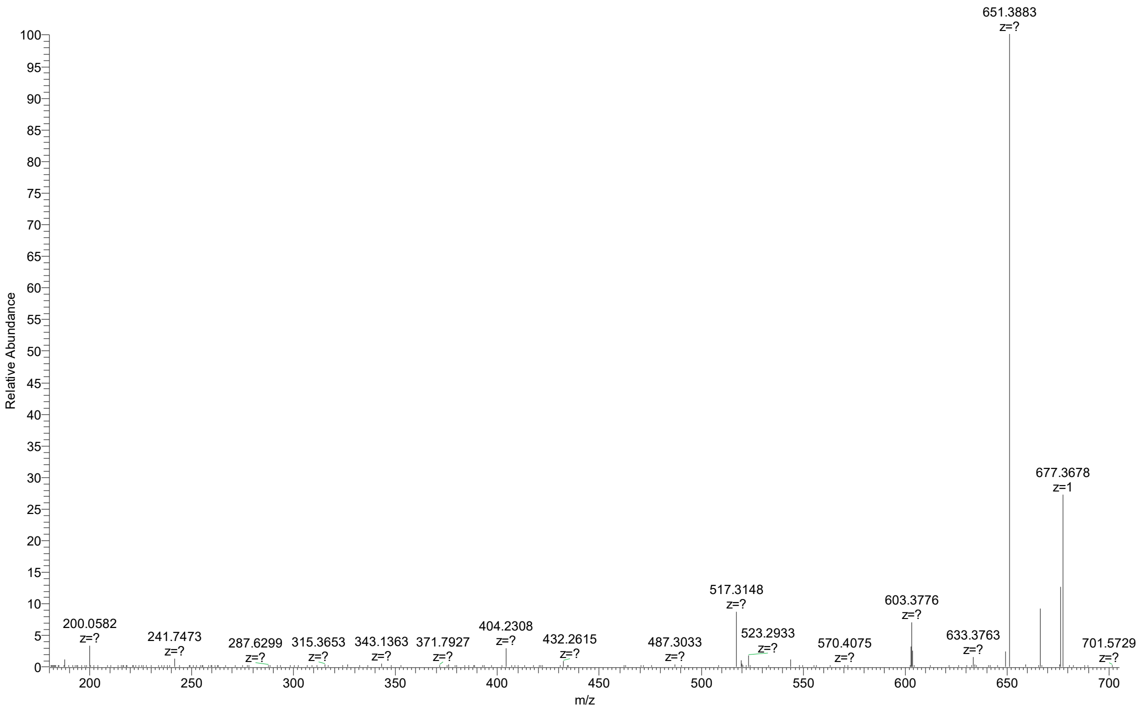

Figure S16. HRMS/MS spectrum of AP-AV693 (**14**) (*m/z* 694.4003).

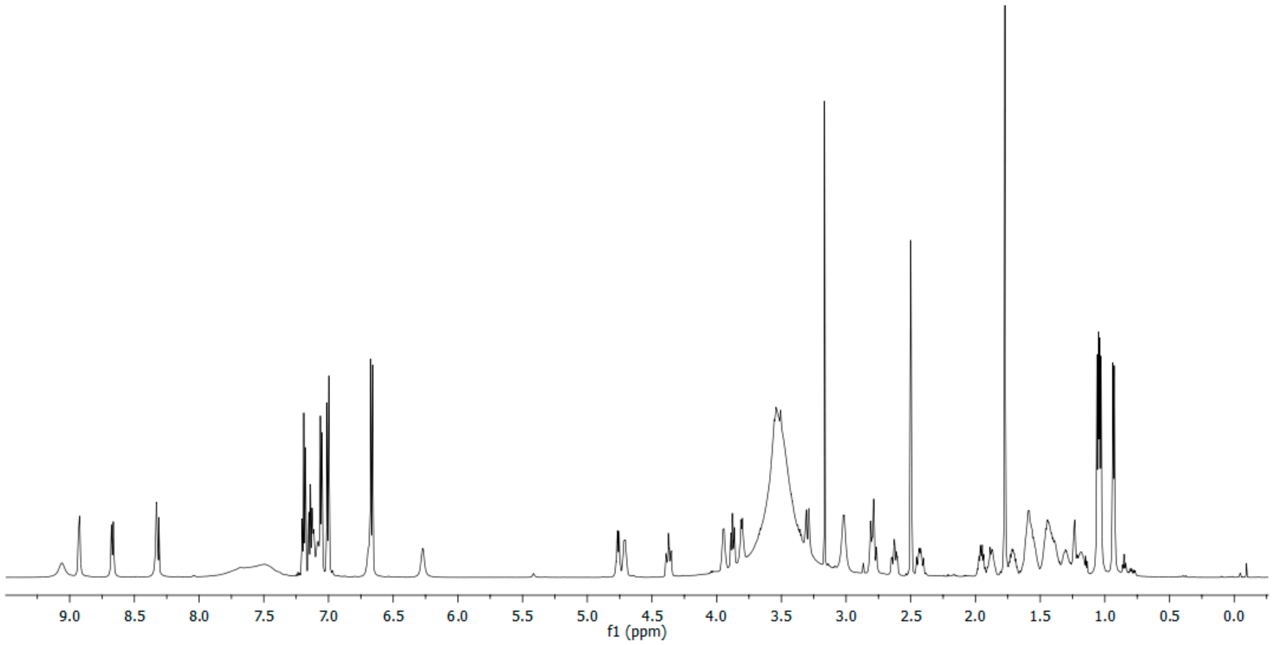

Figure S17. ^{1}H-NMR spectrum of AP-B (**2**) (700 MHz, DMSO-d_6).

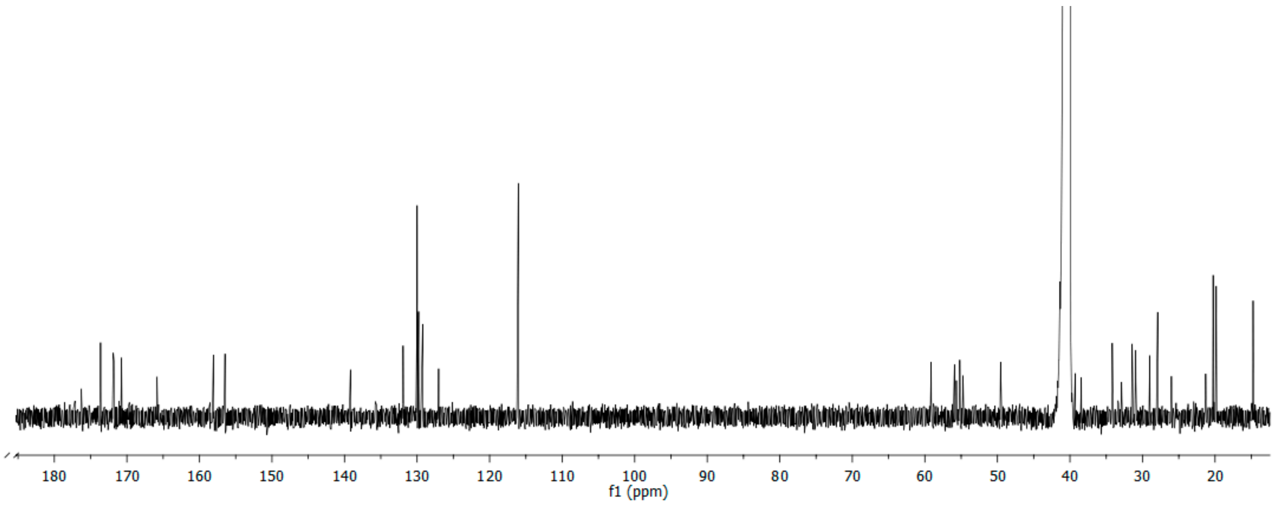

Figure S18. ^{13}C-NMR spectrum of AP-B (**2**) (700 MHz, DMSO-d_6).

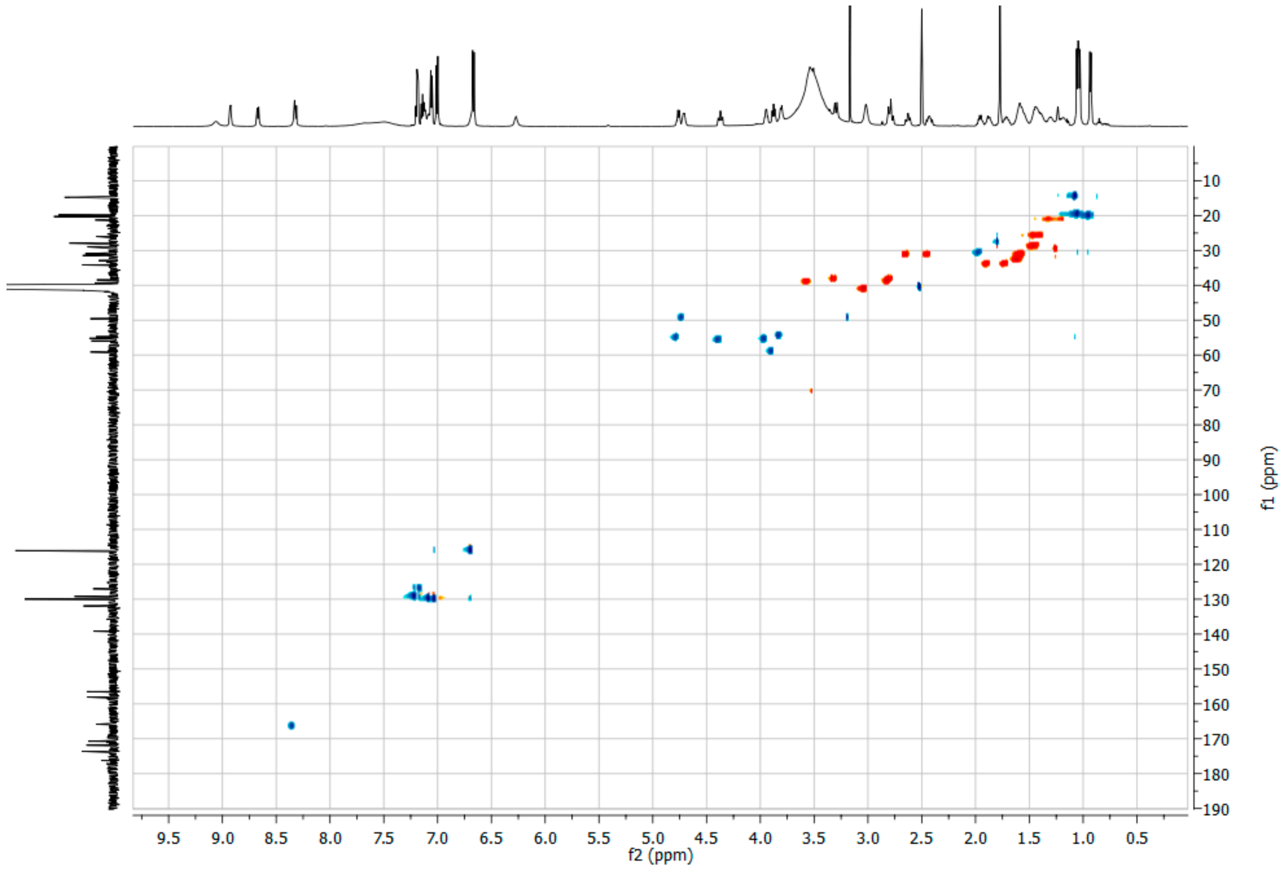

Figure S19. HSQC-NMR spectrum of AP-B (**2**) (700 MHz, DMSO-d$_6$).

Table S1. NMR data of AP-B (**2**) (700 MHz, DMSO-d$_6$).

AA	pos.	δ_C, type		δ_H, mult (J in Hz)
**Phe**	1	170.7, C		
	2	55.9, CH		4.41, ddd (12.5,8.7,3.2)
	3	38.4, CH_2	a	3.59, dd (13.2, 3.2)
			b	2.82, dd (13.2,12.5)
	4	139.2, C		
	5, 9	129.8, CH		7.10, d (7.0)
	6, 8	129.3, CH		7.23, dd (7.4, 7.0)
	7	127.0, CH		7.18, m
	NH*			8.71, d (8.7)
**MeAla**	1	165.9, C		
	2	55.2, CH		4.80, q (6.9)
	3	14.8, CH_3		1.09, d (6.9)
	N-Me	28.0, CH_3		1.82, s
**Hty**	1	171.8, C		
	2	49.5, CH		4.75, m
	3	34.1, CH_2	a	1.92, m
			b	1.75, m
	4	31.4, CH_2	a	2.66, ddd (13.3,10.7,3.9)
			b	2.48, ddd (13.3,10.7,6.8)
	5	131.9, C		
	6, 10	130.1, CH		7.05, d (8.4)
	7, 9	116.2, CH		6.71, d (8.4)
	8	156.5, C		
	NH*			8.97, d (4.0)
	OH*			9.11, br s
**Val**	1	173.6, C		
	2	59.1, CH		3.92, t (7.6)
	3	30.9, CH		1.99, m
	4	20.3, CH_3		0.97, d (6.4)
	5	19.9, CH_3		1.08, d (6.6)
	NH*			7.16, d (7.6)
**Lys**	1	171.9, C		
	2	55.7, CH		3.98, dd (11.6,6.3)
	3	32.9, CH_2		1.6, m
	4	21.3, CH_2	a	1.34, m
			b	1.21, m
	5	29.0, CH_2		1.48, m
	6	41.0, CH_2		3.07, m
	α-NH*			6.67, d (6.3)
	ε-NH*			7.43, m
**Arg**	1	176.3, C		
	2	54.7, CH		3.8, m
	3	30.2, CH_2		1.58, m
	4	26.0, CH_2	a	1.48, m
			b	1.42, m
	5	41.0, CH_2		3.07, m
	6	158.0, C		
	α-NH*			6.27, br s
	δ-NH*			7.43, m
	CO(ureido)	158.1, C		

**These signals were attributed by comparison with literature data.*
